# Infrared Photovoltaic–Battery Hybrid Systems Enabled by Colloidal Quantum Dots

**DOI:** 10.1002/asia.202401958

**Published:** 2025-10-01

**Authors:** Hong Ji, Renjun Liu, Peter Smowton, Bo Hou

**Affiliations:** ^1^ School of Physics and Astronomy Cardiff University Cardiff UK

**Keywords:** CQD, Hybrid, Near‐infrared photovoltaics, Photovoltaic‐battery, PV

## Abstract

Photovoltaic‐battery (PV/B) hybrid energy systems have gained global attention due to the depletion of fossil fuels and environmental concerns. PV systems play a crucial role in sustainable power generation as they produce clean, environmentally‐friendly energy directly from sunlight. Compared to the first‐generation silicon PVs, which are ineffective for wavelengths beyond 1100 nm, and the high‐cost second‐generation III‐V semiconductors, Colloidal quantum dots (CQDs) stand out as a key material in the latest generation of PVs. They offer the advantage of absorbing light in the near‐infrared (NIR) range at a significantly lower cost. Current PV/B systems often integrate silicon PVs with lithium‐ion batteries or direct photobatteries and have demonstrated reliable performance. Although substantial academic work has focused on NIR CQD‐based PVs and batteries, research directions in NIR PV/B hybrid systems remain somewhat disorganized and uneven. This review focus on: (i) providing a detailed analysis of CQDs, including their chemical and physical properties and their applications in NIR PV cells, and (ii) summarizing literature on past and current NIR CQDPVs and batteries that could be used in PV/B systems, synthesizing diverse sources of information. This article seeks to offer new insights and identify potential future research directions for professionals in the field.

## Graphical abstract

Photovoltaic–battery (PV/B) hybrid systems are key for sustainable energy but face cost and efficiency limits. Colloidal quantum dots (CQDs) enable low‐cost near‐infrared light harvesting, surpassing silicon and III–V PVs. This review analyzes CQD properties, their roles in NIR PVs and batteries, and synthesizes current research to highlight progress and future directions in NIR PV/B hybrid systems.

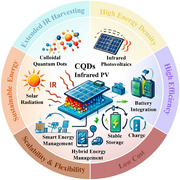

## 1 Introduction

**Figure 1:**
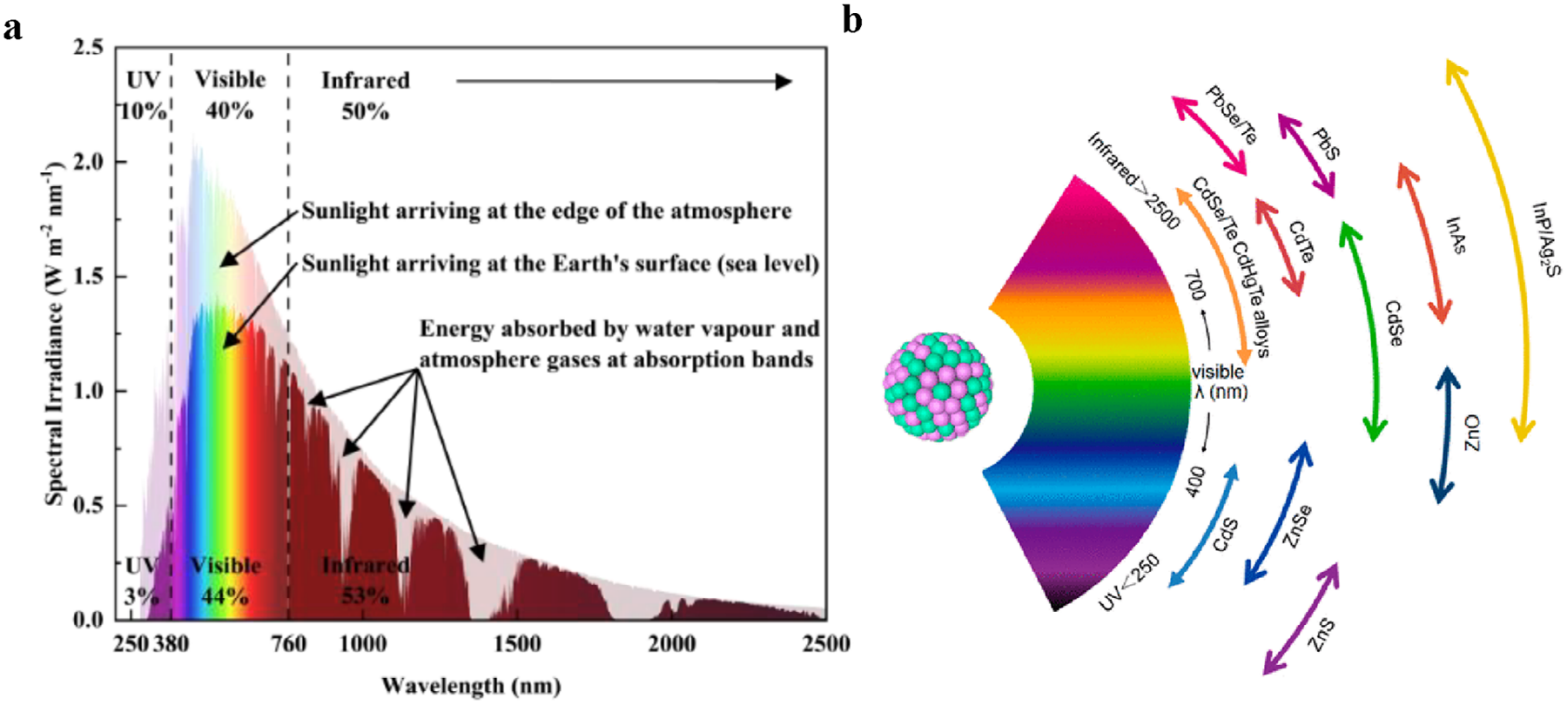
a) Solar radiation spectrum distribution, b) representative CQDs core materials scaled as a function of their emission wavelength superimposed over the spectrum. Reproduced from, Ref. [1] copyright 2018 American Chemical Society.

Solar irradiance in space (depicted by the transparent rainbow curve in Figure 1a) undergoes attenuation as it reaches Earth's surface. This attenuated irradiance, called global tilt irradiance or AM 1.5G, includes both direct and diffuse sunlight and is shown by the solid rainbow curve. The total power density for AM 1.5G is around 100 mW/cm^2^, with a spectral intensity distribution that matches sunlight at Earth's surface at a solar zenith angle of 48.2^○^. The AM1.5G spectrum is a broad spectrum, covering wavelengths from approximately 280 nm in the ultraviolet range up to 4000 nm in the mid‐infrared region.

Figure 1a shows the wavelength solar irradiation spectrum between 280 to 2500 nm. The NIR portion of the solar spectrum (760 to 1400 nm, sometimes extended up to 2500 nm) contributes significantly to the infrared portion of solar irradiance, which collectively accounts for approximately 53% of the total solar irradiance for electricity generation.

At the same time, the penetrability of NIR radiation under atmospheric conditions is more reliable than visible light, particularly under conditions involving fog and clouds, due to reduced scattering and absorption by certain atmospheric constituents such as water vapor, carbon dioxide, dust, and ozone. Thus, it is an emerging technology for the development of NIR solar power due to its reliability and consistency. The traditional silicon‐based solar cell can normally capture light from about 400 to 1100 nm. However, the absorption of silicon drops massively for the wavelength longer than 1000 nm, which prevents its utility for this wider range of the solar spectrum, especially NIR light. Currently, NIR PV technology primarily relies on materials such as Indium Gallium Arsenide (InGaAs), Gallium Arsenide (GaAs), and other compound semiconductors. However, these solar cells face significant drawbacks, including their high cost and relatively low efficiency. This reduced efficiency is attributed to various factors, including lattice mismatch, surface recombination, and challenges with surface passivation.^[^ ^2^, ^3^ ^]^

Colloidal quantum dot (CQD) technology overcomes this limitation. CQDs are nanoscale direct bandgap semiconductor materials with unique optoelectronic properties, which can be dispersed in solvent. They can be designed to absorb light within a specific wavelength range by choosing suitable semiconductor materials with a desired bandgap and specific size as shown in Figure 1b.

This enables QD photovoltaics (QDPV) to more efficiently absorb and utilize a wider range of the solar spectrum from visible light to NIR light, significantly improving the overall efficiency of solar cells. Here are some of the key advantages of QDPVs:

**Spectral tunability**: The band structure of CQDs can be tuned by changing their size and material composition, allowing them to absorb different wavelengths from visible light to NIR light.

**Efficient energy conversion**: CQDs exhibit a phenomenon called multiple exciton generation, which can generate multiple electron‐hole pairs when absorbing one photon, improving the photoelectric conversion efficiency.

**Manufacturing flexibility**: CQDs can be prepared by low‐cost methods such as solution processing and are compatible with existing solar cell manufacturing processes. In this work, the solution processed method will be described.

In general, by addressing the limitations of the traditional silicon‐based solar cells (unable to absorb the light beyond 1100 nm), the overall efficiency and reliability of QDPVs are significantly improved, especially under complex atmospheric conditions. This makes NIR solar technology an important development direction in the future of solar power generation.

The capabilities and advantages of QDPVs in capturing and converting a broader spectrum of solar energy make it crucial to consider how this technology can be integrated into the broader renewable energy sector, especially the combination with advantage battery storage. By coupling QDPVs with state‐of‐the‐art battery technologies the intermittent nature of solar power can be stabilized, storing excess energy produced during peak sunlight hours for later use during periods of low solar irradiation or high demand. Thus, enhancing the efficiency and reliability of power supply systems in diverse environmental conditions.

Grid‐tied and off‐grid are the two most commonly used applications for PV/B hybrid energy systems. A grid‐tied PV/B system is connected to the local utility grid, providing power to buildings and storing excess energy, which helps reduce dependence on nonrenewable sources and lower costs by feeding excess power back. It enhances grid stability and provides backup power during outages. Although off‐grid PV/B systems operate independently, ideal for remote areas, achieving energy independence by generating, storing, and managing power without external support, ensuring a consistent and reliable energy supply under various conditions while reducing expense.

The versatility and transformative potential of QDPV solar systems make them crucial in global efforts toward sustainable energy solutions. Whether enhancing power supply performance and resilience or providing essential power in rural locations, QDPV solar systems are at the forefront of the renewable energy revolution. The NIR PV/B energy system is also advantageous in space applications due to its broader light absorption range, enhanced energy capture in low‐light conditions, temperature tolerance, and lightweight and flexible design. These features make it a consistent, efficient, and reliable energy source, which is crucial for the success of space missions.

The implementations of the QD PV/B systems, emphasizing system design considerations, are described in the following sections. We start with the introduction of the current NIR CQD materials that hold potential for such systems and outline the most commonly used battery materials. Finally, the paper will address the challenges associated with PV/B systems, with a particular focus on efficiency and safety concerns.

## 2 Fundamentals of CQDs and QDPV

CQDs are nanometre‐scale semiconductor particles that exhibit unique chemical and physical characteristics, determined by quantum confinement at the nanoscale, affecting their optoelectronic properties and broad‐spectrum absorption. A range of synthesis techniques allow for customization of CQD size, shape, and surface properties to improve photovoltaic efficiency. Moreover, understanding the mechanisms behind CQD performance in QDPV, such as charge separation, transport, and extraction, is essential for advancing solar cell efficiency.

### 2.1 Composition and Properties of CQD

#### 2.1.1 Chemical Properties

CQD are nanoscale semiconductor particles with the diameter typically between 2 and 10 nm. These nanoparticles usually consist of a semiconductor core made from Group II–VI (e.g., cadmium selenide (CdSe), cadmium sulfide (CdS)) or Group III–V (e.g., indium phosphide (InP), and GaAs) materials.^[^ ^4^ ^]^ The nature of these materials greatly affects the electronic properties such as bandgap. Apart from the basic core structure, some CQDs are designed with an additional semiconductor covering to form a core‐shell structure. This shell material usually has a wider bandgap than the core and serves to further passivate the core surface, separating carriers from surface defects to improve optical properties such as photoluminescence (PL) efficiency and stability.

**Figure 2:**
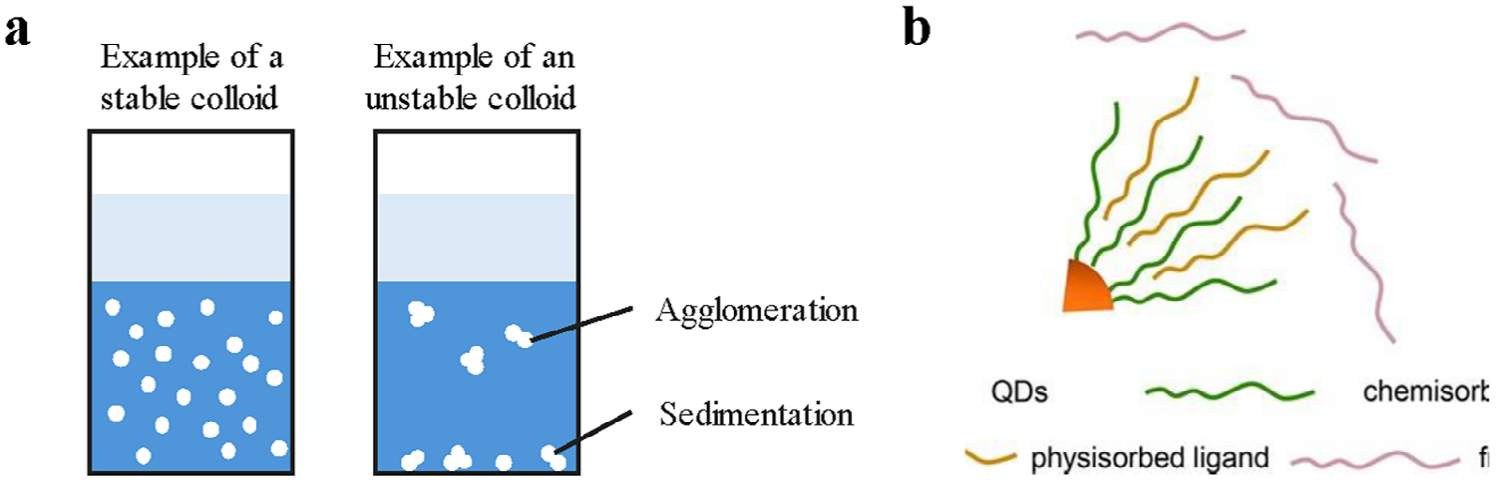
a) Stable and unstable colloids, b) illustration of the forms of the ligands present on the surface of CQDs. Reproduced from, Ref. [5] copyright 2020, American Chemical Society.

Typically, the surface of these nanoparticles is passivated with organic ligands. These molecules can bind the surface atoms of the CQDs and stabilize the CQDs from oxidation, agglomeration, or sedimentation, and therefore form a stable colloid in the solution. Figure 2a illustrates the difference between stable and unstable colloid.

Figure 2b represents the forms of the ligands present on the surface of CQDs. The choice of ligands affects the solubility, stability, and interaction of CQDs with other materials. Effective passivation by appropriate ligands is also critical during device fabrication, as it ensures high quantum efficiency and charge carrier mobility.

**Figure 3:**
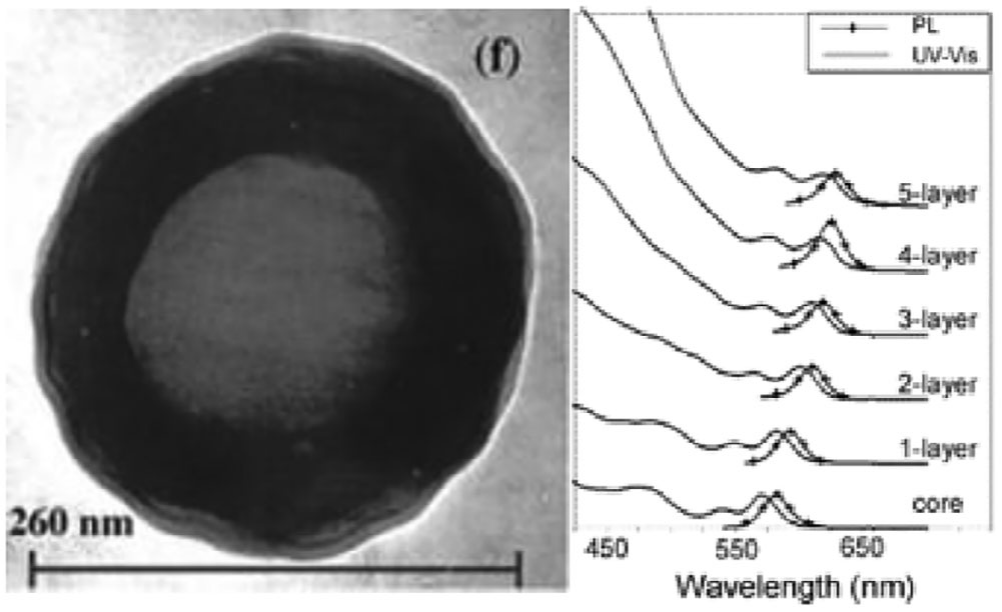
Left: The core/shell structure. Reproduced from, Ref. [6] copyright 1999 American Chemical Society. Right: The evolution of the UV–vis and PL spectra of the core/shell nanocrystals upon the growth of subsequent atomic layers of the CdS shell. Reproduced from, Ref. [7] with the permission from Copyright 2003 American Chemical Society.

In a core‐shell structure, as shown in left side of Figure 3, the shell typically acts as a bridge to help confine excitons within the core. This configuration allows for more effective photon absorption and improved exciton transfer to the photoactive layers of the device. As a result, recombination losses are minimized, and charge carrier mobility between CQDs is enhanced. In subsequent steps, this structure can influence properties such as absorption and PL, further optimizing the device's performance as shown in right part of Figure 3.

All these parameters collectively define the chemical properties of CQDs and influence their optical and electronic properties, solubility, stability, and compatibility.

#### 2.1.2 Physical Properties

One of the most distinctive physical properties of CQDs is the quantum confinement effect, which refers to their size‐dependent electronic and optical properties. The quantum confinement effect is observed when the size of the CQDs is smaller than the exciton Bohr radius.^[^ ^8^ ^]^ It was first experimentally observed in 1974 when Dingle et al.^[^ ^9^ ^]^ studied different quantum size structure, which can be classified based on the number of dimensions in which electrons and holes have free motion. Interestingly, they found that in a 0D structure (quantum dots), the manifestation of this structure is reflected in the shift of the absorption band edge and the luminescence maximum as the average size of the semiconductor nanoparticles changes. Efros et al.^[^ ^10^ ^]^ theoretically substantiated the quantum confinement effect in 1982 and then it was experimentally demonstrated in 1985 by A. I. Ekimov^[^ ^11^ ^]^ on the CdS and CuCl nanocrystals whose size was from 1.2 to 32 nm and 2 to 31 nm. In such small nanoparticles, the movement of electrons and holes is more tightly restricted, requiring more energy to bridge the gap between the valence band and the conduction band. Small CQDs interact with photons of short wavelengths (high energy), whereas large CQDs absorb and emit photons with longer wavelengths (low energy).

**Figure 4:**
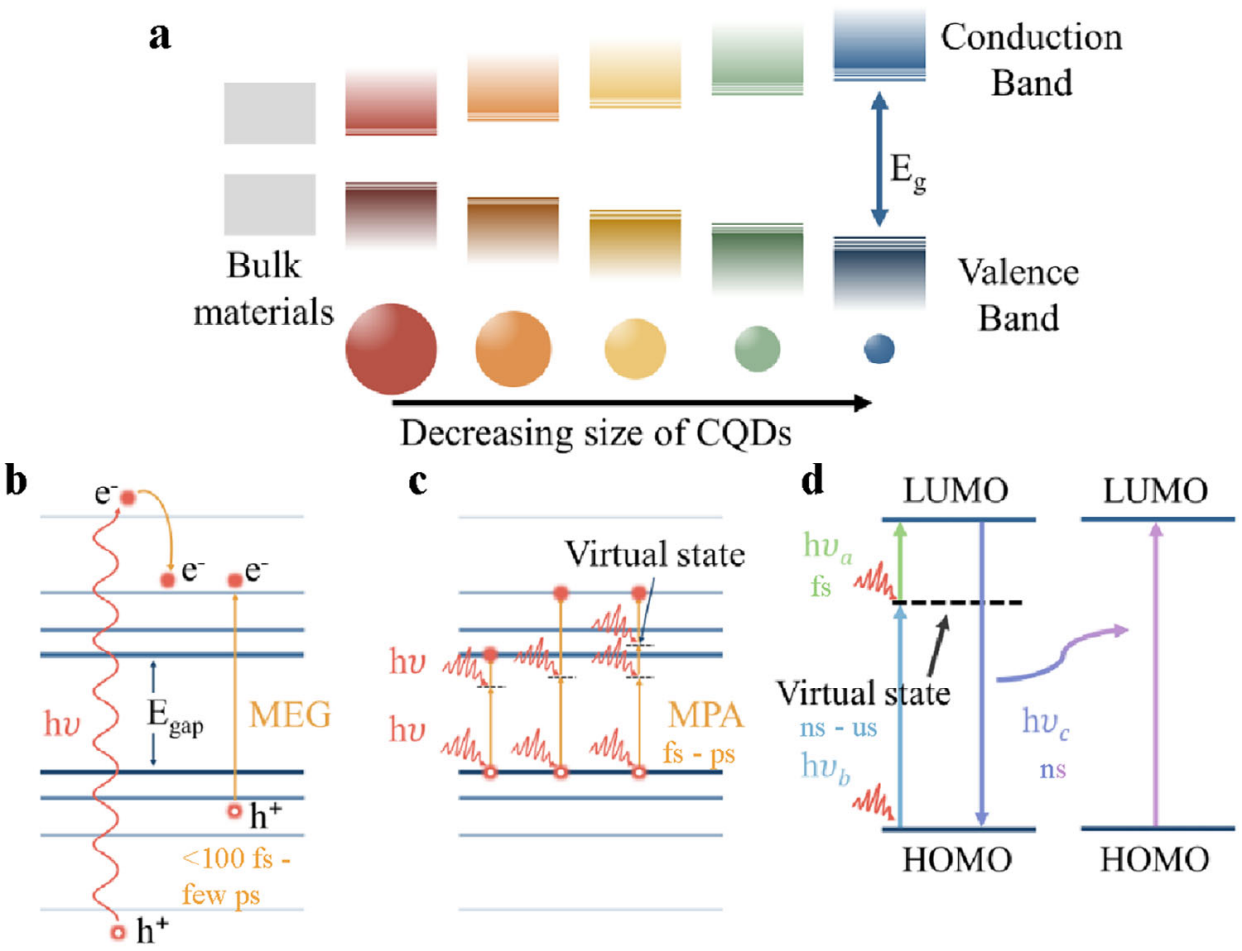
a) Size‐dependent quantum confinement‐enabled band gap tunability from CQDs, b) schematic diagram of multiple exciton generation (MEG), c) schematic diagram of multiple photon absorption (MPA), and d) schematic diagram illustrating upconverted photoluminescence.

The tunability of the bandgap is shown in Figure 4a. By varying CQD size, it allows for precise control of solar spectrum absorption, which is particularly valuable in photovoltaics. By incorporating CQDs of various sizes into a single solar cell, a broad‐spectrum photovoltaic device capable of absorbing light from ultraviolet to near infrared can be achieved.

Another remarkable feature of CQDs is multiple exciton generation (MEG) (Figure 4b) and multiple photon absorption (MPA) (Figure 4c). MEG refers to the phenomenon that enables them to generate multiple excitons from a single high‐energy photon, bypassing hot‐carrier cooling via photon emission.^[^ ^12^, ^13^, ^14^, ^15^, ^16^ ^]^

The mechanism of this phenomenon was first proposed in 2001 and was observed experimentally 3 years later.^[^ ^17^, ^18^ ^]^ In bulk semiconductor materials, when a photon with energy higher than the bandgap is absorbed, the excess energy typically causes vibrations within the lattice and is dissipated as heat. However, with MEG, CQDs can potentially capture this excess energy to produce multiple charge carriers, offering the potential to surpass the Shockley–Queisser limit and enhance the overall conversion efficiency of photovoltaics. While MPA represents a nonlinear optical process in which two or more photons of lower energy are absorbed simultaneously by a CQD material, the combined energies equal or exceed the bandgap, enabling the excitation of an electron to a higher electronic state.^[^ ^19^ ^]^ The right side of the Figure 4c illustrates the representative diagram for MPA.

Upconverted photoluminescence (UCPL) refers to the phenomena of PL with photon energies higher than the energy of an exciton photon, which is also called blue‐shift luminescence. This process is highly linked with MPA. In this process, two or more low‐energy photons are absorbed, they can collectively elevate electrons to a higher energy state, leading to emission at a shorter wavelength. Figure 4d illustrates the change of the state of the molecule.

Photon absorption through a virtual state allows the molecule to reach an excited state, the lowest unoccupied molecular orbital (LUMO) from where it can radiate back giving rise to the photoluminescence. LUMO and HOMO (the highest occupied molecular orbital) shown in Figure 4d stand for the lowest unoccupied molecular orbital and the highest occupied molecular orbital, respectively. UCPL can positively affect the efficiency of the solar cell with a better photon utilization.

#### 2.1.3 Relevance to Photovoltaics

These additional mechanisms allow CQDs to absorb a wide range of the solar spectrum, improving the ability to harvest light. The application of tandem solar cell is designed to capture a broader spectrum of sunlight by integrating various CQDs with different bandgap. The extended wavelength collection will enhance the EQE and increase the PCE. By utilizing different bandgap Lead Sulfide (PbS) CQDs into devices Hou et al.^[^ ^20^ ^]^ achieved a cascaded‐junction QDPV with the maximum PCE of 9.05%, which is higher than any of its constituent QDPVs.

CQDs can also be combined with organic or perovskite photovoltaics. Kim et al.^[^ ^21^ ^]^ investigated the hybrid organic/CQDs to enhance device performance. The best PCE of tandem structure of 7.9% is better than either PbS QDPV of 4.8% or OPV of 6.6%. Manekkathodi et al.^[^ ^22^ ^]^ used solution‐processed perovskite and CQDs to enhance infrared light collection beyond 1000 nm, and achieved a PCE of over 20%.

The high absorption coefficient of CQDs allows a large amount of light to be captured even in a thin layer, which is ideal for creating lightweight and flexible solar cells. A summary of the absorption coefficient of different dimensions of materials including bulk, molecules, CQDs, nanowires, and quantum wells is summarized in Table 1

**Table 1:** A summary of the absorption coefficient of different dimensions of materials.

Material dimension	Description	Absorption characteristics	Examples	Absorption co‐efficient	Refs.
Bulk materials	Three‐dimensional structures with continuous electronic bands.	Absorb light over a broad range of wavelengths due to the continuous electronic bands; absorption coefficients depend on material composition and structure.	Gallium arsenide (GaAs)	∼10^4^	^[^ ^23^, ^24^ ^]^
			Silicon (Si)	∼10^3^	^[^ ^25^ ^]^
Molecules	Two or more atoms chemically bond together.	Exhibit sharp absorption peaks corresponding to specific electronic transitions; absorption spectra are highly specific to molecular structure.	Chlorophyll	∼10^5^	^[^ ^26^ ^]^
			Rhodamine B	∼10^6^	^[^ ^27^ ^]^
Quantum dots (0D)	Nanoscale particles with quantum confinement in all three dimensions.	Display size‐dependent absorption spectra; smaller CQDs absorb at shorter wavelengths (blue shift), while larger CQDs absorb at longer wavelengths (red shift). High molar absorption coefficients due to discrete energy levels.	CdSe	∼10^4^	^[^ ^28^ ^]^
			PbS	∼10^5^	^[^ ^29^ ^]^
Nanowires (1D)	Structures with quantum confinement in two dimensions.	Show anisotropic absorption properties; absorption spectra can be tuned by varying diameter and material composition.	GaAs nanowires	Highly size‐dependent	^[^ ^30^ ^]^
			Niobium nitride (NbN) nanowires	Highly size‐dependent	^[^ ^31^ ^]^
Quantum wells (2D)	Thin layers confining charge carriers in 1D.	Exhibit step‐like absorption spectra; absorption edges can be tuned by adjusting well thickness and material composition.	InGaAsP quantum wells	Varies with well width and composition	^[^ ^32^ ^]^
			MgZnCdSe quantum wells	Varies with well width and composition	^[^ ^33^ ^]^

The absorption coefficient units for bulk materials usually expressed in cm^−1^, representing the attenuation of light per unit length as it travels through the material. For the quantum wells, absorption can be characterized by the absorption coefficient per unit length. But due to the confined nature of these structures, the absorption may also be described per well or per unit area. However, for the molecules, CQDs, and nanowires materials, the absorption coefficient units usually expressed in M^−1^cm^−1^ (where M denotes molarity), which is related to concentration and path length.

The absorption coefficient μ for a dispersion of CQDs as:^[^ ^34^, ^35^ ^]^

$$\mu =\frac{2\pi }{\lambda n_{s}}\left|f_{\mathrm{LF}}{|}^{2}f\varepsilon_{1}=\frac{n}{n_{s}}\right|f_{\mathrm{LF}}{|}^{2}f\alpha$$

By comparing μ (the absorption coefficient of the composite) with the absorption coefficient α of the corresponding bulk material, it becomes clear that the local field effect significantly modifies the absorption of a material when it is dispersed as spherical particles within another medium. The symbols used and the quantity they represent are listed in Table 2.

**Table 2:** Overview of often used symbols, the quantity they represent and their SI units.

Symbol	Quantity	SI units
λ	The given wavelength	m
*n_s_*	Refractive index of the solvent	–
*f_LF_*	Local field factor	–
*f*	Volume fraction	–
ε_1_	Imaginary part of the dielectric function	–
α	Absorption coefficient of a homogeneous bulk material	m^−1^

**Figure 5:**
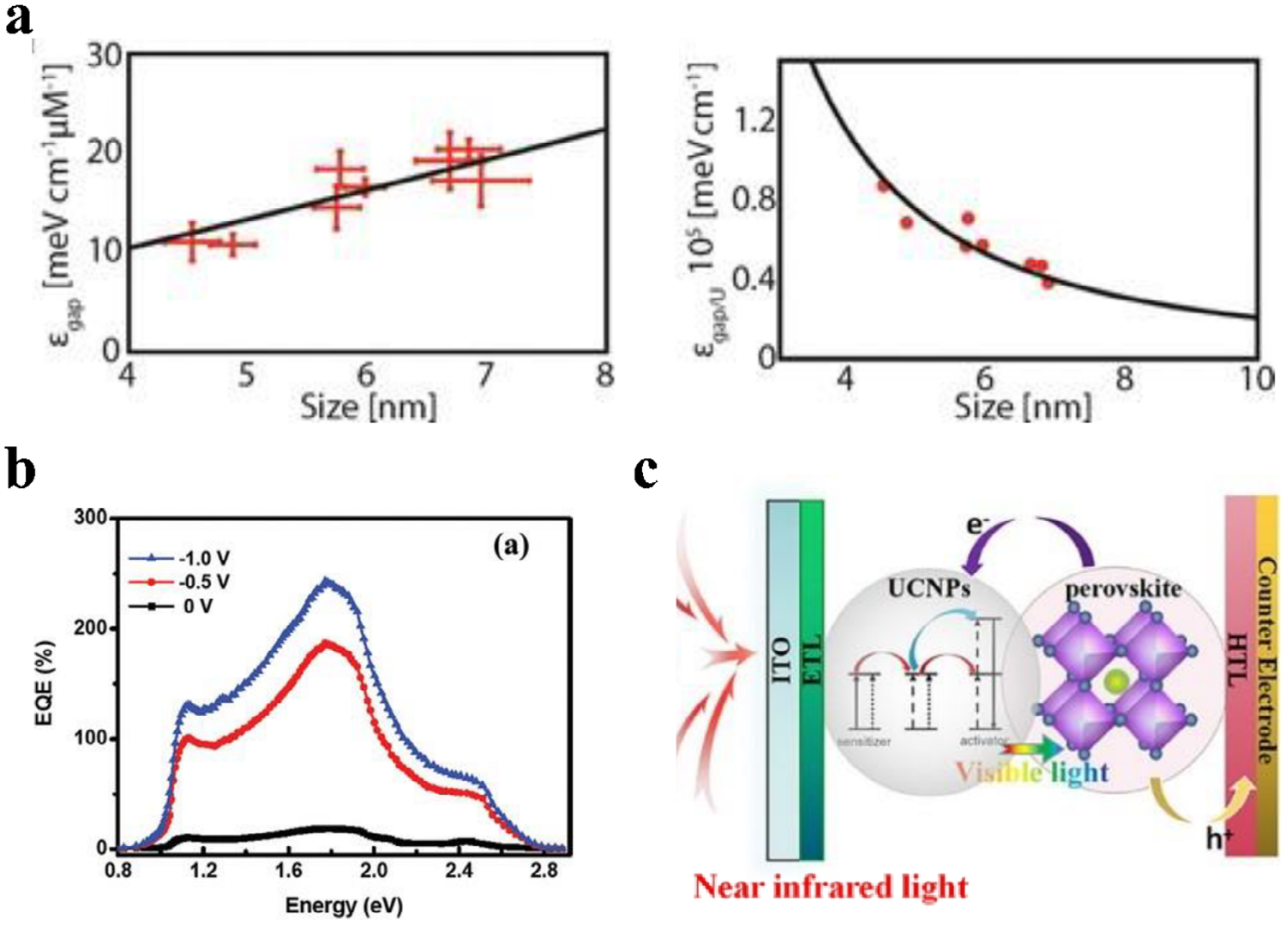
a) Absorption coefficient of PbTe NCs at the band gap. Reproduced from, Ref. [36] copyright 2019 American Chemical Society, b) EQE curves. Reproduced from, Ref. [39] copyright 2012 American Chemical Society, c) scheme of the energy transfer process in PV. Reproduced from, Ref. [40] copyright 2021 Elsevier Ltd.

Figure 5a shows the absorption coefficient of PbTe NCs of different sizes.^[^ ^36^ ^]^ The relation obtained from data in Figure 5a is similar compared to the energy‐integrated molar absorption coefficients of PbS and PbSe.^[^ ^37^, ^38^ ^]^

Hence, due to the unique large absorption coefficient and size tunability, by utilizing the CQDs with appropriate sizes and thickness, the light absorption can be maximized which can further enhance the PV device performance.

The phenomenon of MEG allows for efficiencies beyond the Shockley–Queisser limit, which is unattainable traditionally. Semonin et al.^[^ ^41^ ^]^ achieved a PbSe QD‐based solar cell with EQE of 114  ± 1% and IQE of 130%. IQE greater than 100% means the CQDs generate more electron‐hole pairs than the number of incident photons absorbed. Manna et al.^[^ ^39^ ^]^ also achieved a Si/CdS nanowire heterojunction photodetector in the visible–near‐infrared spectrum with EQE in excess of 100% as shown in Figure 5b.

UCPL is an important phenomenon that could potentially enhance the solar cell performance, by converting low‐energy photons (such as infrared light) into higher‐energy photons (visible light) that the solar cell can more readily absorb and convert into electricity. Numerous studies have reported UCPL in CQDs. For example, Ouyang et al.^[^ ^42^ ^]^ investigated the UCPL in colloidal CdS and ZnCdS CQDs in the visible light range. Yang et al.^[^ ^43^ ^]^ studied the NIR to visible UCPL by ZnTe/CdSe@CdS@CdSe/ZnSe type II/type I double CQDs. Sun et al.^[^ ^44^ ^]^ reported efficient NIR‐to‐visible UCPL in surface amended InAs CQDs with the passivation by growing an ultrathin ZnSe shell. Liang et al.^[^ ^40^ ^]^ explored the impact of UCPL solar cell performance. Rare‐earth ions were employed with the device structure shown in Figure 5c.

The solution‐processable nature of CQDs enables low‐cost fabrication and scalability, as they can be applied to photovoltaic devices by various deposition methods such as spin coating, inkjet printing, and roll‐to‐roll processing. This versatility aids in the creation of next‐generation, affordable solar cells that can deployed in different applications, including portable or integrated photovoltaic systems.

### 2.2 Synthesis Methods

Various physical and chemical techniques have been developed to synthesize CQDs using either top‐down or bottom‐up approaches.^[^ ^45^ ^]^ The top‐down approach involves breaking down bulk materials into nanoscale particles through methods including chemical exfoliation^[^ ^46^ ^]^ and mechanical exfoliation,^[^ ^47^ ^]^ such as electrochemical,^[^ ^48^ ^]^ chemical oxidation,^[^ ^49^ ^]^ ultrasonication,^[^ ^50^ ^]^ and laser ablation.^[^ ^51^ ^]^ In contrast, bottom‐up methods assemble nanoparticles from fundamental building blocks, such as atoms and molecules, using methods like hot injection,^[^ ^52^ ^]^ microwave,^[^ ^53^ ^]^ thermal decomposition,^[^ ^54^ ^]^ solvothermal synthesis,^[^ ^55^ ^]^ chemical precipitation, and biosynthesis.^[^ ^56^ ^]^

**Figure 6:**
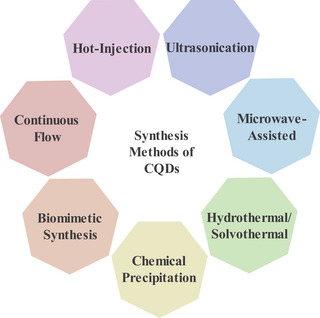
Common synthesis methods for CQDs.

This study will introduce six widely used methods for synthesizing CQDs, as illustrated in Figure 6.

#### 2.2.1 Hot‐Injection

**Figure 7:**
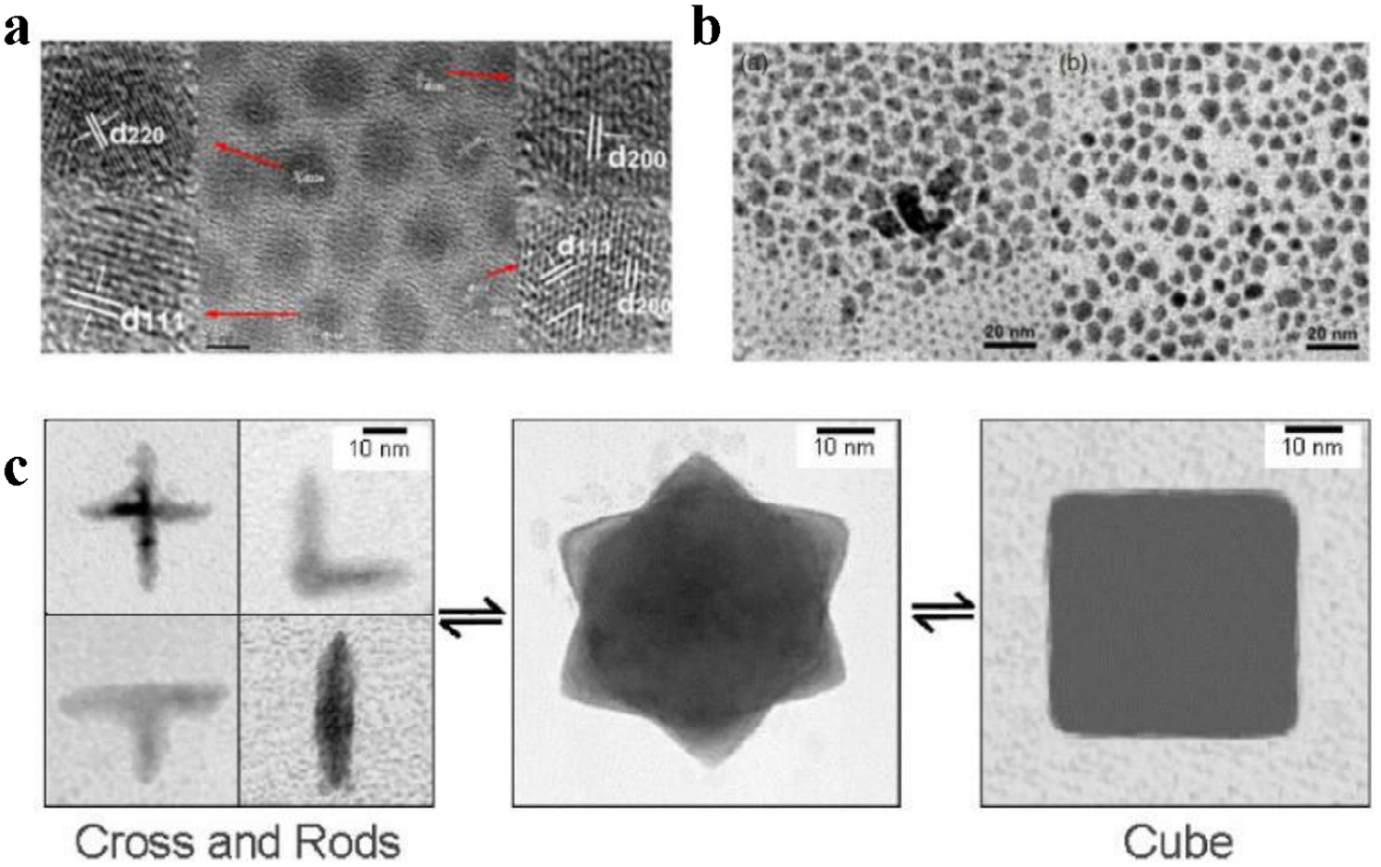
a) Self‐assembly of CQDs with highly monodispersed size. Reproduced from, Ref. [20] copyright 2016 American Chemical Society, b) TEM images of PbS nanocrystals (reaction under RT and 100 °C). Reproduced from, Ref. [60] copyright 2002 American Chemical Society, c) TEM image of PbS nanocrystals synthesized at different temperatures. Reproduced from, Ref. [60] copyright 2002 American Chemical Society.

Murray, Norris, and Bawendi first proposed the hot‐injection technique in 1993 to produce CQDs,^[^ ^52^ ^]^ which has since become a widely used method. This approach initially used to synthesize A^II^B^VI^ semiconductor CQDs was extended to other types of CQDs such as A^III^B^V^ and A^IV^B^VI^. This method relies on rapid nucleation in a temperature‐controlled environment with primary advantage of its capability to easily tune the CQDs size.^[^ ^57^, ^58^ ^]^ In this process, a precursor solution, in which metal ions or organometallic compounds are dissolved in a solvent, is quickly injected into another high‐temperature precursor solvent, leading to a rapid reaction that produces the desired CQDs. By altering the concentration of the precursor, reaction time, and temperature, the size and composition of CQDs can be adjusted.^[^ ^59^ ^]^ This approach can generate high‐quality CQDs with a homogeneous size distribution (shown in Figure 7a) in an efficient and quick way.

The hot‐injection method is particularly useful for the synthesis of inorganic CQDs, such as Pb(S,Se), halide perovskites, ZnSe, InP, Cd(Se,Se), and Cu_2_ZnSn(S,Se)_4_ etc.^[^ ^61^, ^62^, ^63^, ^64^, ^65^ ^]^ M.A. Hines and G.D. Scholes reported that the synthesis of PbS CQDs was carried out under both the “Hot Injection Method” (HI) and the “Room Temperature Method” (RT) as shown in Figure 7b.^[^ ^66^ ^]^

It was observed that the CQDs produced via the HI approach have a strongly angular and faceted shape, whereas the RT method resulted in smoother particle shapes, as indicated by the decreased number of sharp edges on the particles. Cheon and co‐workers demonstrated that by varying the injection temperature, the shape of the resulting particles can be tuned from crosses and rods to multipods to cubes, by varying the ratio of the precursor, the shape evolves from nearly spherically shaped tetradecahedrons to almost cubic. This provides more insight into the shape evolution of PbS nanocrystals during ripening observed with the HI method as shown in Figure 7c.^[^ ^60^ ^]^

A significant amount of data on CQDs has been generated by both experiments and theoretical simulations. Machine learning techniques are applied to analyze and as a feedback mechanism to help the synthesis.^[^ ^67^ ^]^ For example, E. H. Sargent and coworkers incorporate machine learning to analyze the experimental data and propose experimental parameters to try, and, ultimately, point to regions of parameter space that will enable record‐monodispersity PbS quantum dots.^[^ ^68^ ^]^ Based on the strategy supplied by the machine learning (temperature and precursor amount), a record‐large‐bandgap PbS (611 nm exciton) was synthesized and improved monodispersity achieved.

Yang et al. created an autonomous black‐box system to manage CdSe quantum dot synthesis in a microfluidic reactor.^[^ ^69^ ^]^ Using the global search algorithm SNOBFIT, they identified the ideal injection rate and reaction temperature, which enhanced emission intensity at a particular wavelength.^[^ ^70^ ^]^

In general, by adjusting the material, the capping agents, the amount of the precursor, reaction time and reaction temperature, the size, morphology, and composition of colloidal dots can be precisely controlled. These parameters considerably affect the physical, optical, and electrical characteristics of semiconductor nanoparticles compared to their bulk counterparts.^[^ ^71^, ^72^, ^73^, ^74^, ^75^, ^76^ ^]^

#### 2.2.2 Ultrasonication

Ultrasonic refers to a high‐frequency sound wave (usually higher than 22 kHz). The synthesis approach involves continuous ultrasonic waves creating alternating pressure waves within the liquid, initiating flow and forming tiny vacuum bubbles, known as cavitation nuclei.^[^ ^77^ ^]^ Using the rapid formation and implosion of these nuclei will lead to the high‐speed movement of the liquid, forming a strong fluid shear force and create extreme local conditions, such as temperatures of thousands of Kelvin and pressures reaching hundreds of atmospheres. This enables chemical reactions that may not otherwise occur under standard laboratory conditions.^[^ ^77^ ^]^

This approach enables rapid synthesis of CQDs, often within minutes to hours, compared to traditional heating methods. At the same time, the process runs at a relatively low temperature overall, as the energy required is supplied by cavitation nuclei. This reduces energy use and limits thermal degradation. Moreover, by tuning the sonification parameters (such as the frequency, power, and time), the size, crystallinity, and shape of the CQDs can be controlled.

Suslick et al. first proposed and researched on the application of sonochemistry for synthesizing semiconductor quantum dots (CQDs) in the 1990s and early 2000s.^[^ ^78^ ^]^ They recognized the potential of ultrasonic synthesis in creating nanostructured semiconductors with precise size control. One of the earliest CQDs synthesized by the ultrasonic method were cadmium‐based CQDs (like CdS and CdSe).^[^ ^79^, ^80^ ^]^ Around this time, some other researchers such as A. Gedanken also began exploring sonochemistry for synthesizing CQDs like CdS and ZnS.^[^ ^81^, ^82^, ^83^ ^]^

**Figure 8:**
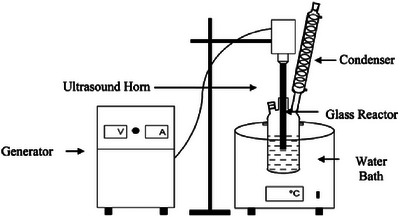
Schematics of experimental setup for ultrasound assisted synthesis of catalysts. Reproduced from, Ref. [84] copyright 2014 American Chemical Society.

In the procedure of ultrasonic synthesis for colloidal CQDs, a solvent that can support sonication containing metal ions or organometallic compounds is prepared. Then subjected to ultrasonic irradiation using a probe or bath sonicator as shown in Figure 8. The cavitation nuclei caused from the ultrasonic irradiation cause rapid heating and cooling, allowing the nucleation of quantum dots.

Based on the application of ultrasonic solubility treatment, this technique can serve as an auxiliary method for preparing CQDs in conjunction with other approaches. Through ultrasonic‐assisted coprecipitation, 0.1 M of Pb(NO_3_)_2_ and 0.1 M Na_2_S were added into a mixed toluene/ water solvent and subjected to an ultrasonic device with frequency of 35 kHz, power output of 300W, and temperature of 20–22 ​°C for 30 mins. Rosiles‐Perez et al.^[^ ^85^ ^]^ successfully produced PbS CQDs with a controlled average size of 6.8 nm. The synthesized PbS CQDs are applied to TiO_2_ photoelectrodes in solar cells through electrophoretic deposition, achieving stable, uniform layers. The cells, with added ZnS for stability, showed a power conversion efficiency of 0.71%.

#### 2.2.3 Microwave‐Assisted Synthesis

**Figure 9:**
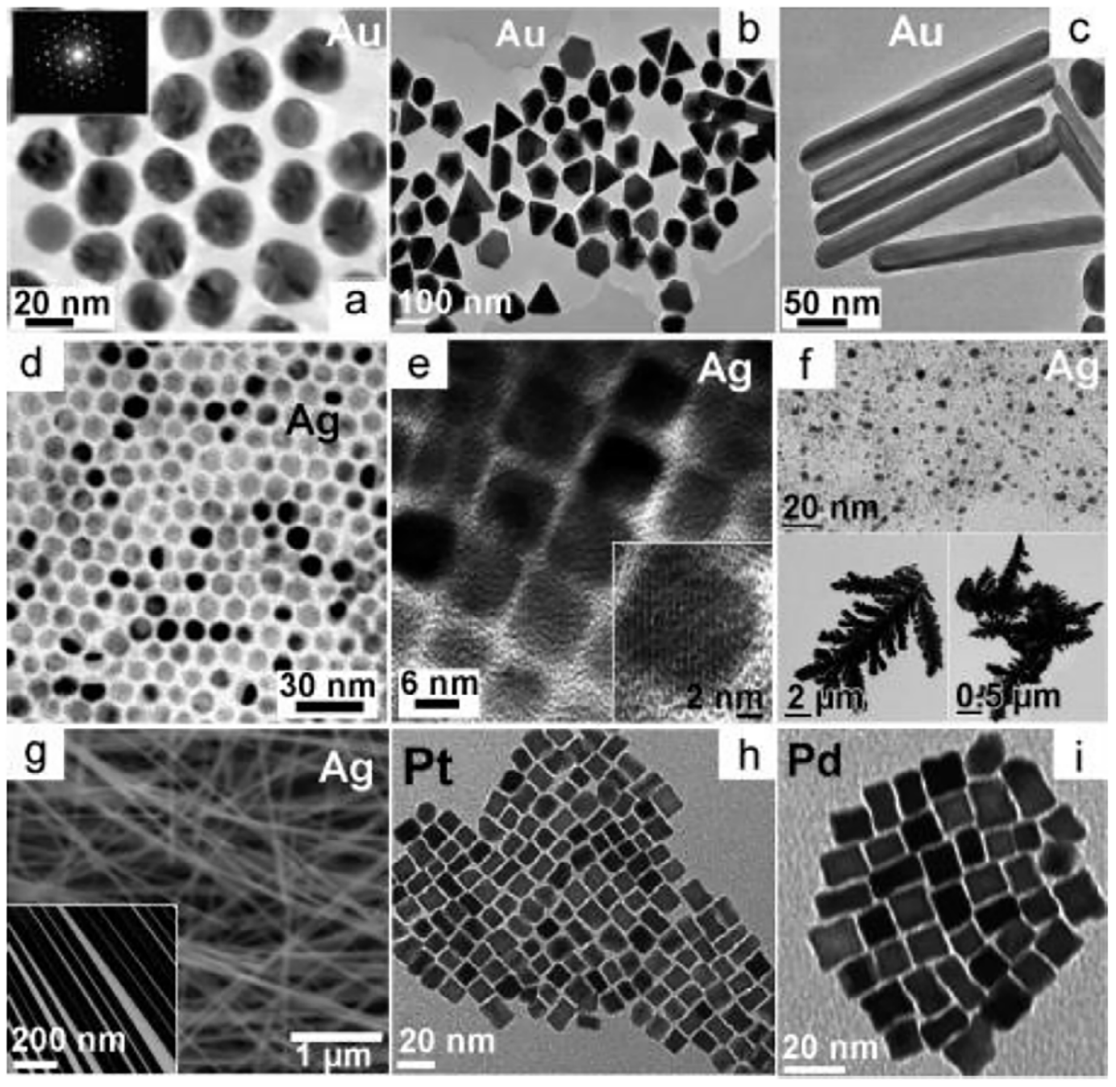
Examples of single‐composition metal NCs grown under MW exposure. Reproduced from, Refs. [88, 89, 90, 91, 92] copyright 2005, 2007, 2008, and 2009 American Chemical Society.

Microwave‐assisted synthesis was first introduced into organic chemistry in the 1986.^[^ ^86^ ^]^ Then Boxall et al. reported this approach for the formation of colloidal Au nanoparticles in 2001.^[^ ^53^, ^87^ ^]^ Synthesis takes place rapidly under an air atmosphere and elevated temperatures and avoids the variation in heating rate associated with conventional batch synthesis. In this approach, a mixture of metal precursor and solvents is exposed to microwave radiation that oscillate at high frequencies (typically around 2.45 GHz).^[^ ^88^ ^]^ When the microwaves pass through polar molecules with electric dipole moment, which are present in certain solvents or metal precursors, the oscillation and rotation of polar molecules generates a large amount of friction to enhance the temperature of materials. Unlike conventional heating, which transfers the heat from the outside to the inside, microwave heating is even and rapid. This uniform heating avoids the effect of temperature variation on the distribution of CQDs, enabling controlled particle nucleation and growth, resulting in consistent sizes and shapes. By varying parameter results can vary as shown in Figure 9, the shapes of same material NCs can be spherical, prismatic, rod, or cubic.

The growth rate can be controlled by reactant concentration and microwave power and the particle size can be controlled by adjusting the reaction temperature and time. This synthesis method has proven to be especially versatile and reliable by A. L. Washington and G. F. Strouse,^[^ ^93^ ^]^ with a low standard deviation in particle size (6% for CdSe and 12% for CdTe) with a reaction time of under 3 min, along with emission line widths around 27–28 nm for CdSe and 40 nm for CdTe.

Zhang et al. investigated the influence of reaction time on CQDs,^[^ ^94^ ^]^ it turned out the color of the solution changed and the size of CQDs varied from 2.75 to 3.65 nm, with the highest PLQY of 6.3%. Different precursors and ligands also affect the optical properties of CQDs. Liu et al. found the choice of precursors will affect the color and PLQY of CQDs,^[^ ^95^ ^]^ the color obtained appeared blue, yellow, and orange with PLQY of 14%, 45%, and 7.5% with the change of phenylenediamine precursor. Duran et al. changed the ligands of CdSe/ZnS CQDs and found out the PLQY of 30%, 25%. and 23%, respectively.^[^ ^96^ ^]^

#### 2.2.4 Hydrothermal/Solvothermal Synthesis

The term ‘hydrothermal’ is originally from geology and describes the formation of various rocks and minerals resulting from water under elevated temperature and pressure. In broad term, this synthesis method is pressure‐ and temperature‐driven, favoring crystalline growth over time.

In 1839, R. W. Bunsen successfully grew barium and strontium carbonate at temperatures above 200 °C and pressures above 100 bars.^[^ ^97^ ^]^ Work on processing of fine to ultrafine particles with a controlled size and morphology started in the 1990s.^[^ ^98^ ^]^

**Figure 10:**
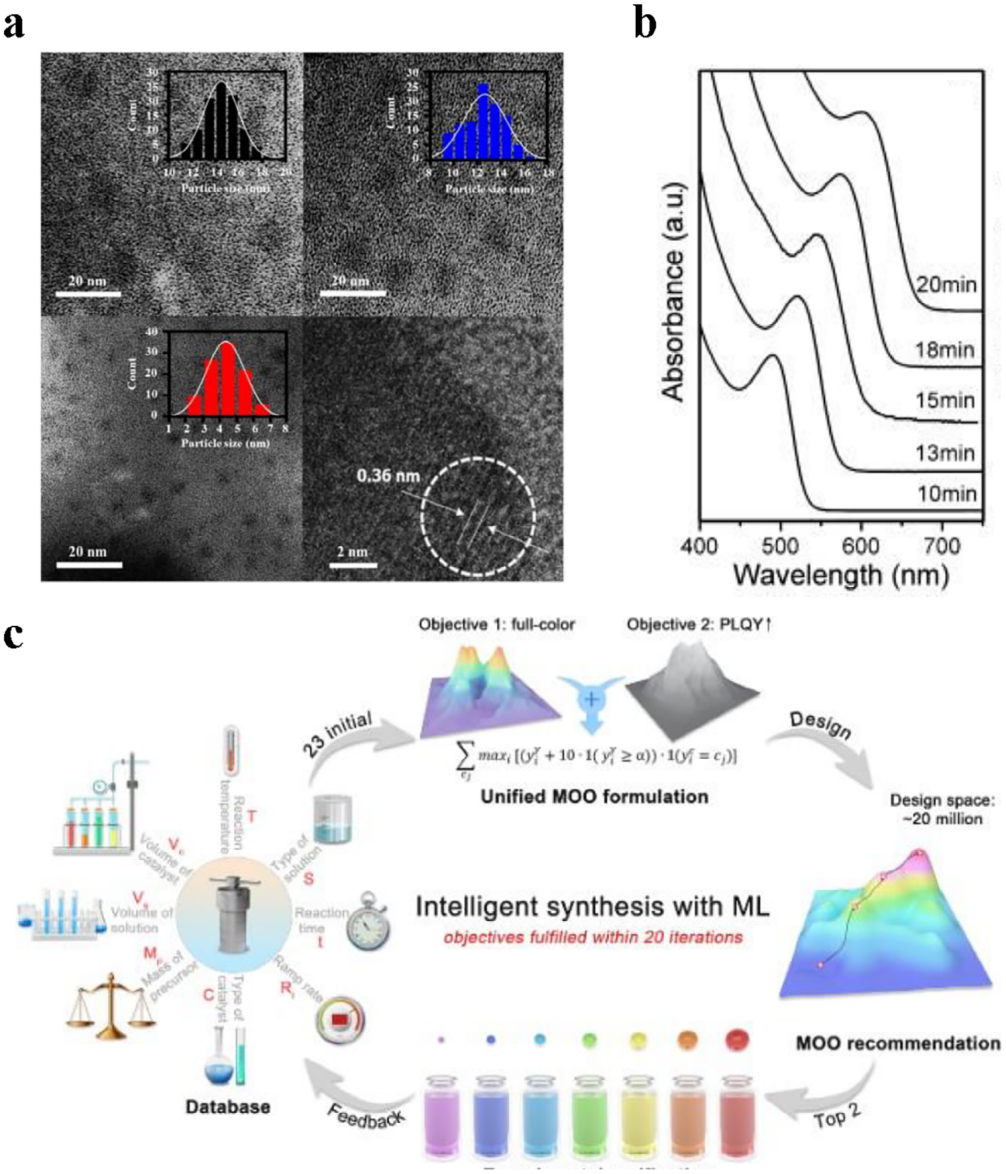
a) TEM images of the CQDs with distinct size distributions. Reproduced from, Ref. [99] b) Temporal evolution of absorption spectra of CA‐capped CdTe CQDs prepared at 220 °C. Reproduced from, Ref. [101] copyright 2008 Elsevier B.V, c) workflow of ML‐guided synthesis of carbon quantum dots with superior optical properties. Reproduced from Ref. [102].

The process is to prepare crystal growth and phase formation by heating aqueous solutions of reactants in a sealed pressure vessel called an autoclave.^[^ ^98^, ^99^, ^100^ ^]^ As the temperature and pressure increase, the precursor solution with metal ions and ligands in this container reaches a supersaturated state and initiates the nucleation of CQDs. By varying the temperature, pressure, and reaction time, the size and shape of the CQDs can be controlled.^[^ ^100^ ^]^ This approach allows for the controlled growth speed, high purity (as shown in Figure 10a), unique morphologies, and relatively low temperatures.

Gao et al. introduced a ligand‐assisted hydrothermal method for synthesizing uniform, monodisperse SnO_2_ CQDs with a narrow size range of approximately 3.6 nm, with a 4.23 eV band gap.^[^ ^55^ ^]^ This relies on the combined use of butyric acid and butylamine as capping agents, which provide particle stability and allow for precise size control. Through the use of the hydrothermal method, the reduction of SnO_2_ size led to an increase in band gap resulting from an upshift of the conduction band minimum band edge position (−3.72 eV) due to the quantum confinement size effect. It should be noted that the increase of band gap is mainly due to the upshift of the conduction band edge with negligible energy level variation from the valence band edge.^[^ ^55^ ^]^

For solar cell applications, Shreya et al. explored the hydrothermal method to synthesize the WS_2_/WO_3_ nanocomposites, by changing the ratio of tungsten and sulfur.^[^ ^103^ ^]^ The optimized hydrothermal synthesis increased optical absorption, enhanced colloidal stability, and reduced charge transfer resistance, with a band gap range of 1.54 to 1.68 eV.

Yang et al. reported the synthesis of cysteamine (CA)‐capped CdTe by hydrothermal method, achieving a high quantum yield (QY) of 19.7%.^[^ ^101^ ^]^ This approach accelerates CQDs growth, resulting in narrow size distribution and high photoluminescence. The CQDs pH‐stability stability occurs at 220 °C. Additionally, as shown in Figure 10b, the UV–vis absorption spectra transferred from 490 to 640 nm and the mean particle sizes evolved from 2 to 4 nm, with reaction time from 10 to 20 min.

Hydrothermal synthesis is widely used for carbon CQDs. Recently, a green hydrothermal method to synthesize carbon CQDs by using lime peels as the carbon source was reported by Gonzalez‐Martinez et al.^[^ ^104^ ^]^ This method achieves carbon CQDs with an average size of 3 nm, ranging from 1 to 6 nm, demonstrated photothermal properties, pH sensitivity, and biocompatibility.

Some recent advances in the hydrothermal synthesis of CQDs include the use of machine learning. Guo et al. developed a multiobjective optimization (MOO) framework to help identify synthesis conditions with full‐color photoluminescence and high quantum yields, leading to a decrease in the experiments that were needed compared to traditional methods.^[^ ^102^ ^]^ This ML‐guided approach, shown in Figure 10c, achieved high quantum yields over 60% across all colors in only 63 experiments by predicting optimal conditions for desired optical properties through training by the dataset input. At the same time, this study also identifies the correlations between synthesis parameters (such as temperature and reaction time) and CQDs' photoluminescence, offering valuable insights for the development of advanced materials.

#### 2.2.5 Chemical Precipitation

Chemical precipitation describes the process in which solid material is formed from within a liquid phase by the introduction of chemical reactants, such as to induce supersaturation. In this method, a metal and a chalcogen precursor are dissolved in the solvent and the temperature is increased to a specific degree, which aims for the decomposition of precursors and begins the nucleation phase. After nucleation, the temperature is slightly lowered to slow down growth which allows for control over the QD size. This is followed by a quench or cooling down reaction to stop the growth. The temperature, precursor concentration, surfactants, and reaction time will affect the CQDs optical properties and morphology.

Typically, solvents such astoluene and hexane, which are nonpolar in nature, are utilized in cases where hydrophobic ligands are used. Ethanol, methanol and *N*,*N*‐dimethylformamide (DMF) are also commonly used. Ramade et al. precipitated PbS QD using ethanol, for use in near infrared solar cells,^[^ ^105^ ^]^ while Zhang^[^ ^106^ ^]^ et al. precipitated their PbS QD through the use of toluene.^[^ ^106^ ^]^

**Figure 11:**
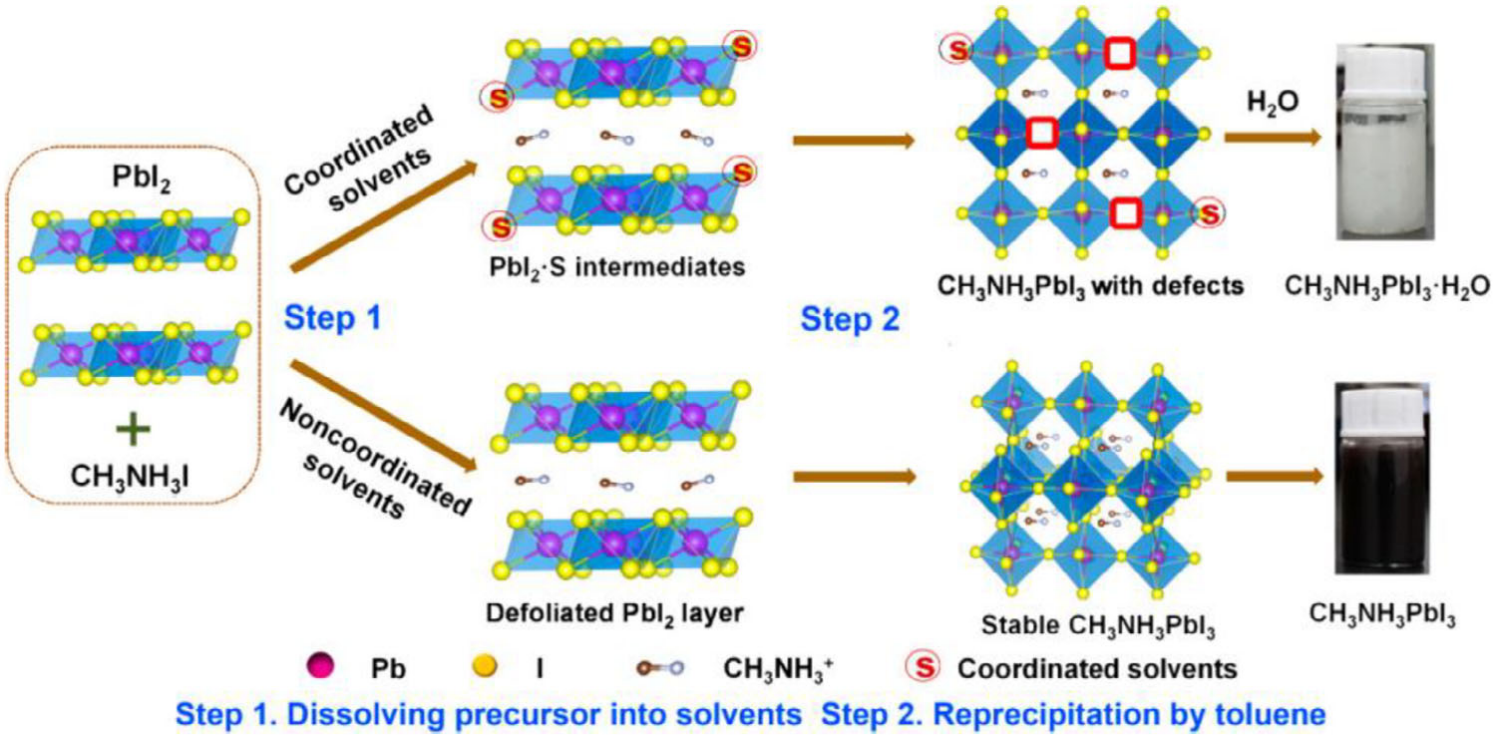
Schematic illustrations of the transformation process. Reproduced from, Ref. [107] copyright © 2017 American Chemical Society.

By applying the strategy of ligand‐assisted precipitation synthesis, Zhang et al.^[^ ^107^ ^]^ successfully invested the challenges of synthesizing air‐stable methylammonium lead iodide (CH_3_NH_3_PbI_3_) CQDs, particularly focusing on improving their stability in ambient conditions with coordinated and noncoordinated ligands, as shown in Figure 11.

They demonstrated that ligands of acetonitrile (ACN) are better than those ligands of DMF and dimethyl sulfoxide (DMSO), as the crystals defects decreased and PLQY achieved up to 46% under ambient conditions with tunable sizes (ranging from 6.6 to 13.3 nm).

Chemical precipitation presents a cost effective, scalable, and reproducible method such as to allow controlled tunability of QD size and characteristics. Within the context of photovoltaic battery sources, this is especially important in cases where large batch sizes quantities are required for device manufacture (roll‐to‐roll processing etc^[^ ^108^ ^]^).

#### 2.2.6 Biomimetic Synthesis

The physical and chemical synthesizing methods have many disadvantages for use in biomedical areas. The poor biocompatibility and low water solubility, make the dots difficult to use directly.^[^ ^109^ ^]^ The advantage of the microbial methods (including bacteria,^[^ ^110^ ^]^ yeasts,^[^ ^111^ ^]^ fungi,^[^ ^112^ ^]^ microalgae,^[^ ^113^ ^]^ and viruses^[^ ^114^ ^]^) of creating CQDs is drawing inspiration from natural processes to improve the synthesis and reduce the presence of toxic materials.

In this method, the metal ions are introduced into microbial environment, then the microbial cells use their metabolic pathways to reduce metal ions by a redox reaction, penetrating and facilitating the formation of CQDs to the outside of the cell. At the same time, the biomolecules such as proteins and amino acids naturally bind with the CQDs after incubation, which supports the stabilization of the CQDs and the enhancement of their compatibility in a biological environment. This approach is especially valuable for the medical and environmental applications.

Dameron et al.^[^ ^112^ ^]^ first reported the fungi‐biosynthesis of CdS CQDs which exhibited better monodispersity compared to those synthesized chemically. Since then, a large number of studies have focused on this area. Based on different precursors, the location of synthesis varies, whether intracellular, extracellular, or on the cell membrane. For example, for intracellular synthesis, Valenzuela‐Ibacata et al.^[^ ^115^ ^]^ synthesized CdS CQDs via bacteria (Escherichia coli) within its cytoplasm, which are stabilized by intracellular proteins. In the extracellular synthesis, silver CQDs can be produced by fungi called Fusarium oxysporum^[^ ^116^ ^]^ extracellularly. For the cell membrane synthesis, Bao et al.^[^ ^117^ ^]^ developed CdTe CQDs via *Escherichia coli*.

**Figure 12:**
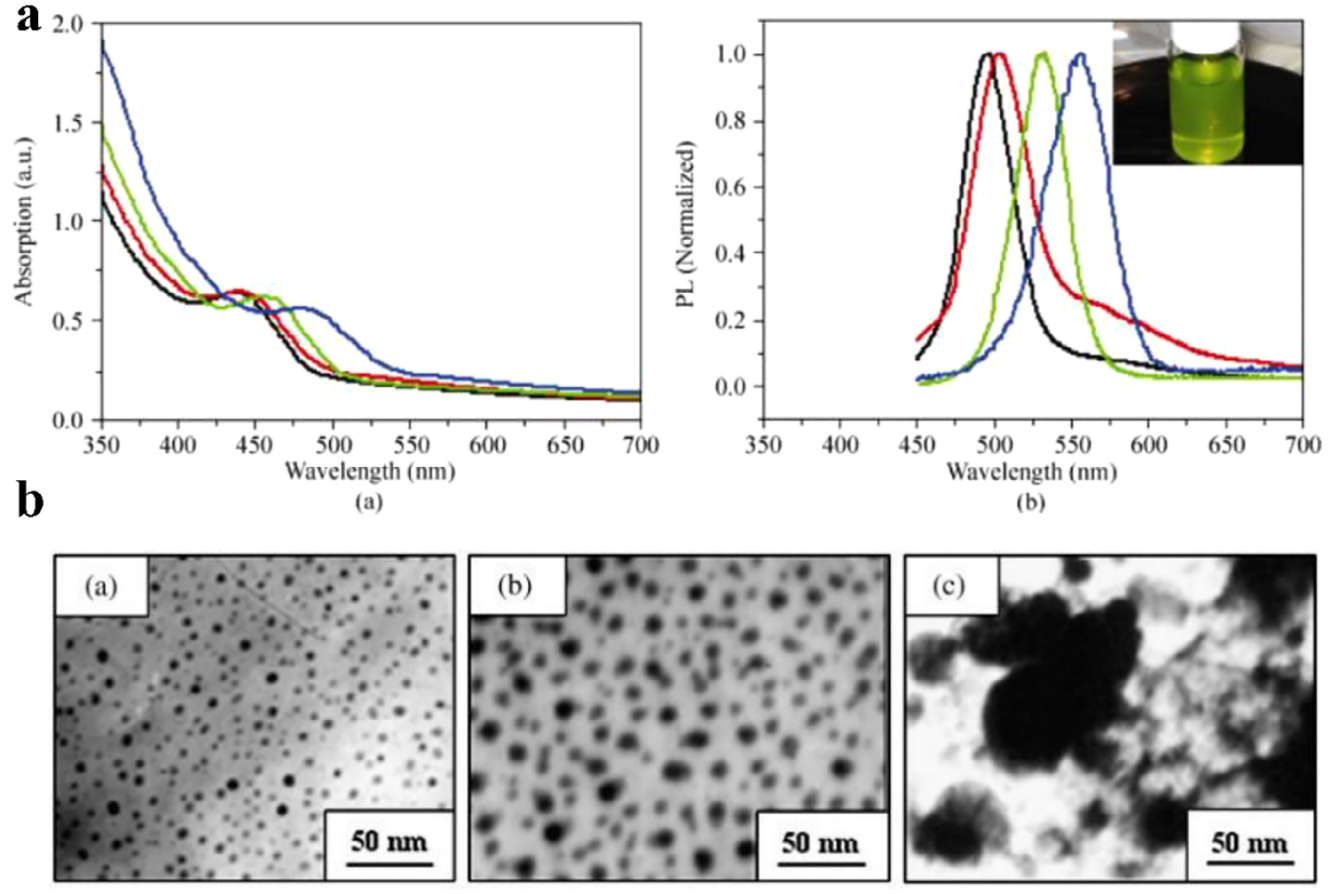
a) UV–vis and PL spectra of the biosynthesized CdTe. Reproduced from, Ref. [118]. b) TEM images of the obtained samples at different culture time. Reproduced from, Ref. [119] copyright © 2009 Elsevier B.V.

Additionally, parameters such as temperature, solution pH, culture time can affect the morphology and optical properties of CQDs. UV–vis and PL spectra of the biosynthesized CdTe CQDs using yeast cells with various reaction times were investigated Bao et al.^[^ ^118^ ^]^ Figure 12a shows the absorption and PL peak shifts as the culture time changed by 1, 2 4, and 8 days.

Bai et al.^[^ ^119^ ^]^ synthesized lead sulfide nanoparticles using Rhodobacter sphaeroides at different culture and obtained PbS with sizes from 10.8 to 3.8 nm as shown in Figure 12b.

The biosynthesis offers significant applicability for producing various CQDs, such as carbon CQDs, metal sulfides, metal selenides, and metal tellurides. Furthermore, this approach aligns with green chemistry principles, as it avoids the need for high temperatures, high pressures, and reaction waste. Nonetheless, further investigation is required to understand the mechanism of crystal nucleation and growth within these microbial reactor synthesis processes.

#### 2.2.7 Continuous Flow Synthesis

In contrast to batch synthesis methods, which perform in discrete steps, continuous flow synthesis requires a flow reactor that allows for the continuous introduction of reactants and collection of products. This strategy was first introduced by Fischer and Giersig for CdS synthesis in 1992,^[^ ^120^ ^]^ and provided enhanced control over reaction parameters and facilitates scalability. In continuous flow synthesis, reactants such as precursors, solvents, and stabilizing ligands are supplied continuously, enabling rapid mixing to achieve homogeneity within microfluidic or tubular reactors. The time the reactants spend in the reactor, known as residence time, plays a crucial role in controlling the nucleation and growth of CQDs. Inside the reactor's reaction zone, parameters such as temperature, flow rate, and pressure are meticulously controlled to ensure the uniformity of size, shape, and composition of the resulting particles. The process concludes with a collection system for product retrieval and postsynthesis treatments.

Moghadam et al.^[^ ^121^ ^]^ pioneered an advanced continuous flow synthesis technique for producing size‐tunable CdSe CQDs with high precision and scalability. They achieved control over CdSe sizes ranging from 3 to 6 nm by adjusting the temperature (240 to 270 °C) and residence time (2 to 20 mins). PLQY of the resulting CdSe CQDs ranged from 11% to 28%. These CQDs exhibited excellent monodispersity and narrow size distributions, with a full width at half maximum (FWHM) of approximately 30 nm. Notably, 167 mg of CQDs with a mean diameter of 4 nm were synthesized in just 87 min. Similarly, Y. T. Didenko and K. S. Suslick^[^ ^122^ ^]^ utilized chemical aerosol flow synthesis to produce CdSe nanoparticles. This method has also been extended to synthesize other CQDs, such as CdS and CdTe, and demonstrates potential for broader applications, including the synthesis of nanostructured metals, oxides, polymers, and various other materials.

In summary, top‐down methods are usually practical for large‐scale production, and bottom‐up methods are ideal for precise control. The reaction conditions, advantages, and disadvantages of the above methods are summarized in Table 3.

**Table 3:** Comparison of some representative synthetic methods according to reaction conditions, advantages, and disadvantages.

Methods	Reaction conditions	Advantages	Disadvantages	Examples	Refs.
Hot‐injection	Heat	Precise size control, high purity, uniform distribution, and low cost	Complex operation, complex equipment, and poor reproducibility	CdSe, PbS, PbSe, ZnS	^[^ ^52^, ^61^, ^123^, ^124^, ^125^, ^126^ ^]^
Ultrasonication	Ultrasound	Simple, green, and large‐scale production	Limited control over size distribution, surface defects, and expensive instrument	CdS, CdSe, ZnS, PbS, ZnO, TiO_2_	^[^ ^79^, ^80^, ^81^, ^82^, ^83^, ^85^, ^127^, ^128^, ^129^, ^130^ ^]^
Microwave	Microwave irradiation	Highly efficient, easy operation, high yield, and scalable	Oxygen defects, expensive instrument	CdSe, CdTe, ZnS, AgInS_2_	^[^ ^93^, ^96^, ^131^, ^132^, ^133^, ^134^ ^]^
Solvothermal	Heat	Low cost, high purity, and green	Low yield, long time, high pressure required	ZnS, CdSe, CdTe, SnO_2_, CuInS_2_	^[^ ^55^, ^101^ ^]^
Chemical precipitation	Controlled pH	Simple, scalable, cost‐effectiveness, and size control	Quality control, environmental and safety concerns	PbS, ZnO, CdS, TiO_2_	^[^ ^105^, ^106^, ^108^ ^]^
Biomimetic synthesis	Microorganism	Green, good universality	Poor size/shape control, immature mechanism	CdS, ZnS, PbS, Ag_2_S	^[^ ^56^, ^135^, ^136^, ^137^, ^138^ ^]^
Continuous flow synthesis	Heat	High scalability, precise control over size and shape, reproducibility, and environmentally friendly	Initial setup complexity, high instrument cost	CdS, CdSe, CdTe	^[^ ^121^, ^122^ ^]^

### 2.3 Mechanism in Photovoltaics

This subsection will provide an introductory framework on the operation of semiconductor P‐N junctions and solar cell devices. Additionally, it will cover bandgap engineering for broad solar spectrum matching and balance between carrier extraction and light absorption in CQD films, aiming to achieve full‐spectrum solar absorption.

#### 2.3.1 Diode Junction (PN, PIN, and Metal Semiconductor Junction)

**Figure 13:**
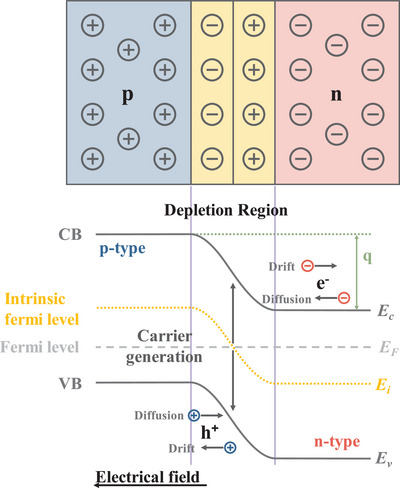
Energy band and schematic of P‐N junction.

A P‐N junction represents an interface or boundary between p‐type and n‐type semiconductor materials, as illustrated in Figure 13. Because of the concentration gradients of holes and electrons across the junction, holes on the p‐side diffuse toward the n‐side, while electrons move from the n‐side to the p‐side, creating a diffusion current. Concurrently, as holes travel to the n‐side, ionized acceptors remain stationary on the p‐side, forming a layer of negative charges in the p‐side of the junction. Similarly, the diffusion of electrons to p‐side leaves behind ionized donors on n‐ side, resulting in the formation of a positive charge layer. This accumulation of opposite charges on either side of the junction forms the depletion region. This charge separation creates an electric field directed from the positive to the negative side, which acts as a barrier preventing further migration of most charge carriers. However, this built‐in electric field also drives electrons from the p‐side to the n‐side in a process known as drift.

The width of the depletion region is:

$$W={\left[\frac{2\varepsilon kT}{q^{2}}\left(\ln\left(\frac{N_{A}N_{D}}{n_{i}^{2}}\right)\left(\frac{1}{N_{A}}+\frac{1}{N_{D}}\right)\right)\right]}^{\frac{1}{2}}$$

$$\varepsilon =\varepsilon_{r}\varepsilon_{0}$$

The depletion region is not symmetrically distributed on both sides; instead, it extends more into the region with lower doping concentration.

$$x_{p0}=\frac{W}{1+N_{A}/N_{D}}$$

$$x_{n0}=\frac{W}{1+N_{D}/N_{A}}$$

The build in potential *V_bi_* reflects the potential change within the depletion region, which is influenced by the doping concentration.

$$V_{bi}=\frac{kT}{q}\ln\left(\frac{N_{A}N_{D}}{n_{i}^{2}}\right)$$

The drift current and diffusion current are (Table 4):

$$I_{drift}=\mu E\tau$$

$$I_{diffusion}=\sqrt{D\tau }$$

A PIN junction is a specialized form of PN junction, distinguished by the inclusion of an intrinsic (undoped) semiconductor layer between the p‐side and n‐side. This design significantly expands the depletion region, enhancing the device's ability to handle high reverse voltages. Most importantly for photovoltaics it extends the volume of the device where absorption and charge separation can occur.

In contrast, a metal‐semiconductor junction occurs where a metal makes contact with a semiconductor material. The properties of this junction are influenced by the work functions of both the metal and the semiconductor (whether doped or undoped). There are two primary types of metal‐semiconductor junctions: Ohmic and Schottky. An Ohmic junction arises when the work functions of the metal and semiconductor are closely matched, enabling current to flow freely in both directions with minimal resistance and exhibiting a linear current‐voltage (I–V) relationship. These junctions are commonly used as contacts in electronic devices for efficient carrier injection and extraction. On the other hand, a Schottky junction forms when there is a significant disparity in the work functions of the metal and semiconductor, resulting in rectifying behavior. For an n‐type semiconductor, the barrier height is given by Φ_*m*_ − χ, while for a p‐type semiconductor, it is calculated as *E_g_* − (Φ_*m*_ − χ), where χ represents the electron affinity, and *E*_g_ is the bandgap of the semiconductor. Early CQD PVs were primarily constructed using a Schottky junction structure. In these designs, CQDs were layered onto a transparent conductive substrate, such as indium tin oxide (ITO) or fluorine‐doped tin oxide (FTO), to establish an ohmic contact. This was followed by the deposition of a metal layer, commonly aluminum (Al), silver (Ag), or magnesium (Mg).^[^ ^139^, ^140^, ^141^ ^]^

The comparison of these three junctions is summarized below Table 5.

#### 2.3.2 Principle of Operation and Fundamentals of PVs

Solar cells operate based on the principle of the photovoltaic effect, which converts photon energy into electrical form. The absorption of above‐bandgap photons by a semiconductor material with a bandgap of *E*_g_ leads to the generation of electron‐hole pairs, as electrons are excited from the VB to the CB. Subsequently, the electric field resulting from the p‐type and n‐type layers in the junction aid in the separation and collection of these electron‐hole pairs. An intrinsic layer, which is an undoped, pure semiconductor material placed between the p‐type and n‐type layers, contains equal numbers of electrons and holes, with the Fermi level positioned midway between the CB and VB, and essentially extends the depletion region. The electric field created by the P‐N junction is now dropped across the intrinsic region as well as the depletion region within the p and n‐type material and separates electron‐hole pairs generated within the extended region. This causes electrons to move to the n‐side and holes to the p‐side, as illustrated in Figure 13.

The PCE is given by:

$$PCE=\frac{V_{m}J_{m}}{P_{in}}=\frac{V_{OC}J_{SC}FF}{P_{in}}$$

where *V*_m_ and *J*_m_ is the voltage and current density at the maximum power point MPP, *P*_in_ is the incident light intensity, *V*_OC_, *J*_SC_, and *FF* is the open circuit voltage, short circuit current density, and fill factor.

The PCE of the PVs based on single‐crystal materials such as silicon and epitaxial compound semiconductors are in the range of 20%–47.1%.^[^ ^148^, ^149^ ^]^ They are limited by the limited spectral absorption range and the considerable exciton dissociation energy.^[^ ^150^ ^]^ At the same time, the cost is usually very high due to the specialized facilities and environmental temperature control and energy consumption required to produce them. Therefore, those semiconductor materials with broad absorption range and cheap fabrication methods, such as solution‐processing and roll‐to‐roll, have attracted more attentions in recent years.

#### 2.3.3 Bandgap Engineering to Match a Broad Solar Spectrum

**Figure 14:**
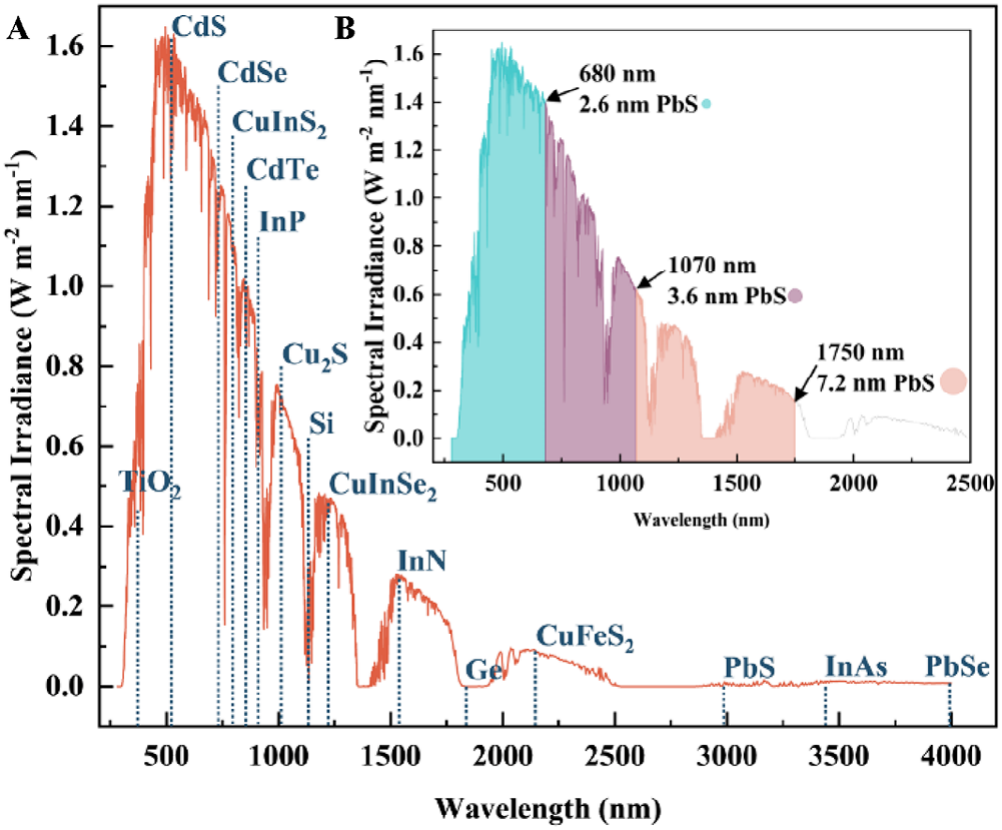
(a) AM 1.5G solar spectrum with bulks semiconductor wavelengths. (b) With different sizes of PbS CQDs.

As shown in Figure 1, near half of the integrated power resides in the IR spectral region. Figure 14a presents the absorption onset for 15 typical bulk semiconductors against the AM1.5G spectrum.^[^ ^143^ ^]^ For inorganic semiconductors, the absorption coefficient typically rises as photon energy increases from the band edge, differing from many organic materials where absorption peaks due to the distinct HOMO‐LUMO transition.^[^ ^151^ ^]^ Low‐bandgap materials absorb more light and generate higher current at a lower voltage, while high‐bandgap materials produce high voltage but lower current due to restricted absorption. Therefore, achieving an optimal PCE in a single‐junction solar cell requires a bandgap between 1.1 and 1.35 eV to balance voltage and current.^[^ ^152^ ^]^ As shown in Figure 14a, bulk materials like InP (1.34 eV), CuS_2_ (with bandgap of 1.21 eV), and Si (1.12 eV) have optimal bandgaps for photovoltaic applications, whereas bulk PbS (0.41 eV), InAs (0.35 eV), and PbSe (0.28 eV) are less suitable.

**Table 4:** Overview of often used symbols, the quantity they represent and their SI units.

Symbol	Quantity	SI units
ε	The permittivity of the semiconductor material	F/m
*k*	Boltzmann's constant	J/K
*T*	Absolute temperature	K
*q*	Elementary charge	C
*n_i_*	Intrinsic carrier concentration	m^−3^
*N_A_*	Acceptor concentration	m^−3^
*N_D_*	Donor concentration	m^−3^
ε_r_	The relative permittivity of the semiconductor	
ε_0_	The permittivity of free space	F/m
*x*_*p*0_	Depletion region width on the p‐side	m
*x*_*n*0_	Depletion region width on the n‐side	m
μ	Mobility of charge carriers	m^2^/(V · s)
*E*	Electric field	V/m
τ	Lifetime of charge carriers	s

One way to tune the bandgap of semiconductor is through compositional adjustment. According to Vegard's law,^[^ ^153^ ^]^ the bandgap of an alloyed semiconductor roughly corresponds to the weighted average of the bandgaps of its constituent materials, as seen in materials like CdSe_1‐x_Te_x_ alloyed CQDs and perovskites.^[^ ^62^, ^154^ ^]^ Although many alloy systems (especially ternary and quaternary alloys) require the use of a bowing parameter to accurately account for the nonlinear dependence of the bandgap on composition, as in InGaN,^[^ ^155^ ^]^ GaNAs,^[^ ^156^ ^]^ and CIGS.^[^ ^157^ ^]^ These materials offer stoichiometrically tailored absorbers that can improve photovoltaic efficiency.

**Table 5:** A summary of different junctions used in CQDs PVs.

Aspect	PN ^[^ ^142^, ^143^ ^]^	PIN ^[^ ^144^, ^145^ ^]^	Metal‐semiconductor ^[^ ^146^, ^147^ ^]^
Formation	p‐type + n‐type semiconductor	p‐type + intrinsic + n‐type semiconductor	metal + semiconductor
Depletion region	Middle size, confined to the interface	Large, covers the intrinsic layer	Ohmic junction: very thin or absent Schottky junction: wide
Behavior	Rectifies current with a threshold voltage	Handles high frequencies, high voltage, and low capacitance	Ohmic junction: linear current‐voltage Schottky junction: rectifying
Current flow	Only in forward bias, reverse bias blocks current	Same as PN junction but with improved performance	Ohmic junction: bidirectional Schottky junction: rectification (allows current in forward bias; blocks in reverse bias)
Advantages	Simple design, good for basic rectification	Better for high‐frequency, high‐power applications	Schottky junction: fast switching, low power loss

However, quantum confinement offers an effective alternative for bandgap engineering without relying on compositional adjustment. By reducing particle sizes to below their Bohr radius, electron and hole wavefunctions become confined, leading to a notable bandgap increase.^[^ ^158^, ^159^ ^]^ This approach allows for the use of low‐bandgap binary compound semiconductors, such as PbS and PbSe, in photovoltaic applications.^[^ ^20^, ^160^ ^]^ For example, as illustrated in Figure 14b, a triple‐junction solar cell can be achieved within a single material system, enabling coverage of an absorption range up to 1750 nm.^[^ ^161^ ^]^ As mentioned before, the highest PCE of 9.05% achieved by Hou et al.^[^ ^20^ ^]^ was accomplished through quantum size effect tuning for the case of PbS. This study involved three different PbS sizes: 4, 3, and 2.31 nm (with bandgaps of 1.03, 1.23, and 1.37 eV, respectively), resulting in cell PCEs that surpassed those achieved by any single‐size PbS QDPV cells.

The drive for solution‐processable materials, combined with the need for broad solar spectrum absorption, has led to significant interest in infrared colloidal nanoparticles. Although materials like GaAs, InP, and CdTe have bulk bandgaps in the NIR range, quantum confinement typically shifts these into the visible spectrum. Although these materials may be valuable in a single junction within a multi‐junction stack, they are unable to span the entire stack due to their limited tunable range, which prevents effective capture of both visible and NIR light simultaneously.^[^ ^161^ ^]^ In contrast, materials with small Bohr exciton radii, such as Si and CuLnS_2_, maintain their bulk characteristics due to minimal quantum confinement effects. As a result, NIR CQDs, which offer broad tunability across the solar spectrum through significant quantum confinement, show great promise for developing low‐cost, high‐efficiency photovoltaics.

#### 2.3.4 Trade‐off Between Carrier Extraction and Light Absorption in CQD Film

The absorption coefficient of silicon is on the order of 10^3^ to 10^4^ cm^−1^, epitaxial semiconductors like GaAs around 10^5^ cm^−1^, and colloidal quantum dots are on the order of 10^5^ to 10^6^ cm^−1^. Therefore, CQDs can capture more light even in a thinner optical layer. However, the trade‐off between the carrier extraction length and optical thickness limits the photocurrent generation and PCE of NIR QD solar cells.^[^ ^162^ ^]^ Based on a conventional experimental method, the ideal thickness of a thin film can be calculated by the absorption coefficient of a thin film covering a transparent semiinfinite substrate, based on the following formula:^[^ ^163^ ^]^

$$d_{film}=\frac{1}{\alpha_{film}}\ln\left(\frac{1-R_{film}}{T_{film}}\right)$$

Here, *R*_film_ and *T*_film_ mean the film reflectance and transmittance. Normally, the completed absorption of IR photons in CQD materials requires thickness on the micrometre range,^[^ ^162^ ^]^ which far exceeds the typical depletion width diffusion length (around 400 nm^[^ ^164^, ^165^ ^]^) of photoexcited charges in CQD materials.

Hence, the ligand exchange and light management strategies have been employed to enhance CQD absorption in the infrared range, aiming to address the absorption–extraction trade‐off.

Light management strategies include the use of multilayer cavities,^[^ ^166^, ^167^, ^168^ ^]^ plasmonic nanoparticles,^[^ ^169^, ^170^, ^171^, ^172^ ^]^ and grating structures.^[^ ^173^, ^174^, ^175^ ^]^ Numerous simulations have been conducted to identify optimal strategies combining various mechanisms to enhance photovoltaic performance. For example, Baitiche et al.^[^ ^176^ ^]^ investigates the enhancement of PCE in an ultrathin solar/thermophotovoltaic cell using a combination of plasmonic metamaterials and arrays. These strategies usually enhance the absorption by increasing the optical path inside the active layers. However, the main drawback of these strategies is the compromise on backside charge collection efficiency and the increase in surface recombination.^[^ ^173^, ^177^ ^]^

Ligand exchange strategies typically involve replacing insulating long‐chain ligands (ex., Oleic acid) with conductive and shorter ligands to enhance carrier transport properties in QD solids, supporting their development for device applications.^[^ ^178^, ^179^, ^180^ ^]^ In solid‐state layer‐by‐layer (LBL) ligand exchange, each layer of a thin film undergoes ligand replacement after CQD deposition. This LBL approach offers precise thickness control and high packing density, essential for effective charge transport and minimized carrier recombination. However, it is time‐intensive and subject to high variability, making control challenging. In contrast, solution‐phase ligand exchange occurs within the solution before CQD deposition, allowing for prefunctionalized CQDs. This method promotes uniform surface passivation, energetic ordering, and compact film formation, significantly enhancing carrier transport properties. For example, in PbS CQDPVs, Hou et al. report a PCE of 9.18% and 10.18% using the LBL approach.^[^ ^20^, ^181^ ^]^ Lan et al. achieved a PCE of 10.6% in a PbS‐CQD cell with solvent‐polarity‐engineered halide passivation.^[^ ^182^ ^]^ Choi et al. attained a PCE of 13.3% through a cascade surface modification strategy,^[^ ^183^ ^]^ while Ding et al. reached a PCE of 15.45% by introducing an organic electron transport layer.^[^ ^184^ ^]^ Nonetheless, further advancements are still being pursued.

**Figure 15:**
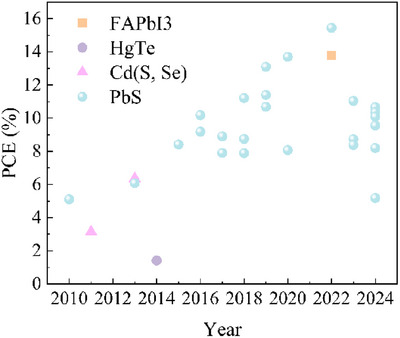
Summary of the photovoltaic performance for NIR CQDSCs.

The PCEs illustrated in Figure 15 provide a comprehensive overview of the advancements in the performance of NIR QDPVs over the last decade. Additionally, Table 6 presents the detailed parameters for each device included in Figure 15, offering valuable insights into the technological progress and characteristics of these photovoltaic devices.

**Table 6:** Detailed parameters of the photovoltaic performance for NIR CQDSCs shown in Figure 15.

year	CQDs	Wavelength (nm)	Semiconducting material	J_SC_ (mA/cm^2^)	V_OC_ (V)	Fill factor	PCE (%)	Refs.
2010	PbS	960	TiO_2_	16.2	0.51	0.58	5.1	^[^ ^185^ ^]^
2011	CdS/Al_2_O_3_/JK‐216	760	TiO_2_	7.186	0.728	0.60	3.14	^[^ ^186^ ^]^
2013	CdSe_0.45_Te_0.55_	800	TiO_2_	19.35	0.571	0.575	6.36	^[^ ^187^ ^]^
2013	PbS	1020	ZnO nanowires	34.47	0.361	0.488	6.076	^[^ ^188^ ^]^
2014	HgTe	950	PSBTBT	6.81	0.45	0.46	1.41	^[^ ^189^ ^]^
2015	PbS	1000	Gold/Silver nanocubes	25.2	0.57	0.59	8.41	^[^ ^169^ ^]^
2016	PbS	1050	ZnO	30.34	0.48	0.63	9.18	^[^ ^181^ ^]^
2016	PbS	905	ZnO	22.6	0.64	0.73	10.18	^[^ ^20^ ^]^
2017	Pbs‐EDT	900	n‐ZnO	12.26	1.13	0.64	8.9	^[^ ^190^ ^]^
2017	PbS / Organic	910	PDPP3T:PC61BM	9.3	1.25	0.672	7.9	^[^ ^21^ ^]^
2018	PbS	1150/1250	ZnO	29	0.5	0.61	8.75	^[^ ^191^ ^]^
2018	Tri‐layer PbS	930	ZnO	26.36	0.615	0.71	11.21	^[^ ^192^ ^]^
2018	PbS/Organic	1350	AZO	33.8	0.41	0.57	7.89	^[^ ^165^ ^]^
2019	PbS‐Al	950	AZO	26.6	0.65	0.66	11.4	^[^ ^193^ ^]^
2019	PbS/Organic	875	PBDTTT‐E‐T	29.6	0.66	0.67	13.1	^[^ ^194^ ^]^
2019	PbS/PbSe	918	ZnO	28.11	0.57	0.663	10.68	^[^ ^160^ ^]^
2020	PbS/Organic	875	ZnO	15.2	1.37	67.7	13.7	^[^ ^195^ ^]^
2020	PbS	1278	ZnO	33.74	0.42	0.57	8.07	^[^ ^164^ ^]^
2022	FAPbI_3_	760	TiO_2_	17.98	1.14	0.66	13.8	^[^ ^196^ ^]^
2022	PbS / Organic	975	ZnO	31.5	0.66	0.743	15.45	^[^ ^184^ ^]^
2023	Mixed nPbS and pPbS	1175/1275	ZnO	36.35	0.45	0.534	8.73	^[^ ^197^ ^]^
2023	PbS/Organosilanes	930	PBDTTT‐E‐T	28.27	0.635	0.615	11.04	^[^ ^198^ ^]^
2023	PbS	1291	ZnO	34.5	0.45	0.54	8.38	^[^ ^199^ ^]^
2024	PbS‐EDT	900	ZnO	26.95	0.64	0.64	10.66	^[^ ^200^ ^]^
2024	PbS‐PbIBr	1300	ZnO	37.32	0.5	0.56	10.36	^[^ ^201^ ^]^
2024	PbS‐I‐Br	890	ZnO	34.54	0.499	0.663	10.09	^[^ ^202^ ^]^
2024	PbS‐I‐Br‐CI	1250	ZnO	35.89	0.47	0.57	9.55	^[^ ^203^ ^]^
2024	PbSe	1270	MXene/ZnO	32.5	0.341	46.6	5.18	^[^ ^204^ ^]^
2024	PbS‐I	980	AZO	27.2	0.59	0.514	8.2	^[^ ^205^ ^]^

## 3 Overview of (Near‐Infrared) Photovoltaic‐Battery Hybrid Energy Systems

**Figure 16:**
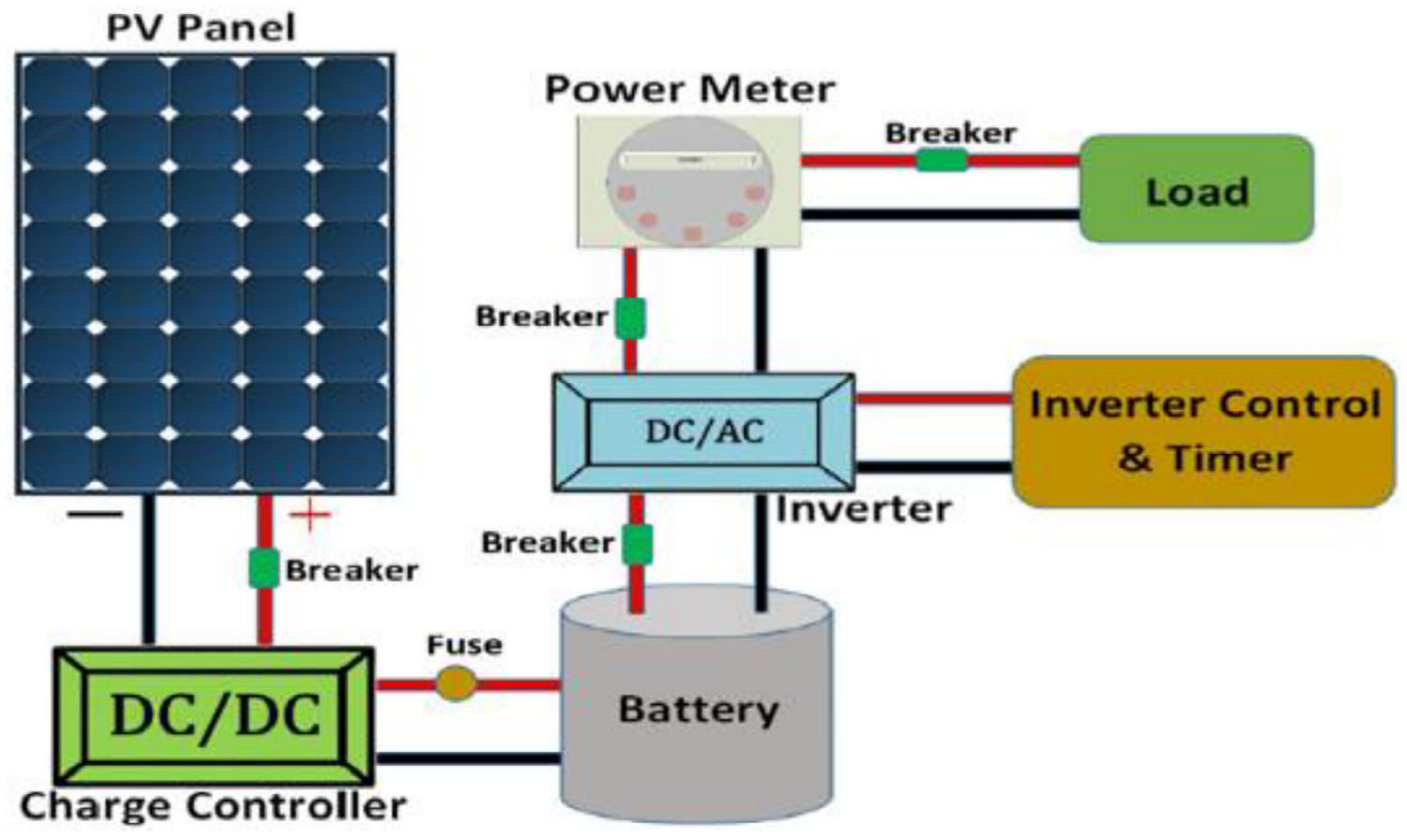
Basic schematic of a PV/B system. Reproduced from Ref. [217].

Over the past two decades, the depletion of traditional energy sources, environmental degradation, and unreliable energy supply have triggered an energy crisis that has attracted significant attention. As a widely used green energy source, solar energy has increased the appeal of photovoltaic‐battery (PV/B) hybrid energy systems, which integrate both PV generation and battery storage components, making them attractive renewable energy solutions. The most straightforward and effective method to harness solar energy is through PV panels, which capture sunlight and convert it into direct current (DC) electricity. This electricity can be stored, used directly, or converted to alternating current (AC) using an inverter. PVs can be classified into three main types based on their semiconductive metals and properties: first‐generation, second‐generation, and third‐generation PVs. Table 7 provides a summary of these types. Figure 16 illustrates a basic PV/B system schematic, showing the solar power source and battery connected to a DC/AC bus through a bidirectional converter, although unidirectional converters may also be used.^[^ ^206^ ^]^

**Table 7:** Summary of the current PV technologies and their applications in PV/B systems.

Categories	First‐generation PV	Second‐generation PV	Third‐generation PV
Raw materials	Polysilicon, Monocrystalline silicon	Amorphous silicon, CdTe, and CIGS	Organic material, Perovskite materials, and Quantum dots
Cost	Highest among all generations	Lowest among all generations	Moderate
Innovation	Oldest and widely used commercially	More recent than 1st generation, well‐developed	Very recent and limited commercial use
Efficiency	Lab‐based 27.3%	Lab‐based 23.6%	Lab‐based 38.8%
Advantages	High PCE	High absorption coefficient, lower cost	Abundant raw materials, simpler fabrication
Disadvantages	Complex fabrication, high cost	Limited raw material availability, potential environmental pollution	Stability issues, durability challenges
NIR range absorption	N	Y	Y
Examples integrated into hybrid system	^[^ ^207^, ^208^, ^209^ ^]^	^[^ ^210^, ^211^, ^212^, ^213^ ^]^	^[^ ^214^, ^215^, ^216^ ^]^

### 3.1 Integration Mechanisms and Design Principles

#### 3.1.1 Overview of Hybrid Systems

**Figure 17:**
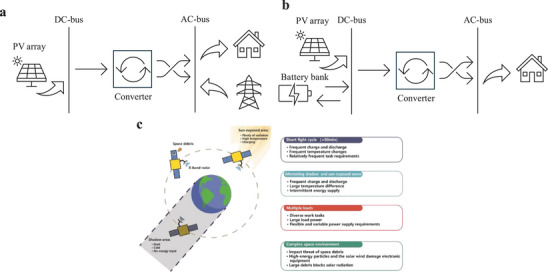
a) Grid‐tied solar PV power system, b) off‐Grid solar PV power system, c) the influencing factors PV/B system faced in space. Reproduced from, Ref. [218] 2022 Elsevier Ltd.

The PV power systems can be categorized into grid‐tied and off‐grid systems based on their connection to the power grid. The grid‐tied, also known as grid‐connected PV system, can operate when interconnected with the utility grids, as shown in Figure 17a. The grid‐tied solar system connects to the local utility grid and can be instrumental in increasing the efficiency and stability of the existing power infrastructure. Grid‐tied QDPV systems can supply electricity to a building or facility and even store surplus solar energy during peak sunlight hours, reducing the load on the local utility grid and minimizing dependency on non‐renewable sources. By feeding excess power generated by QDPV systems back to the grid, electricity costs can be offset. Additionally, this system can provide critical backup power during outages and increase grid stability during fluctuations in power supply or demand.

In contrast, the off‐grid system, also known as stand‐alone PV system, can operate independently, as shown in Figure 17b. An off‐grid solar system does not connect to any local utility grid. This system is typically found in rural or remote areas where traditional grid infrastructure is unavailable or too expensive to install. Off‐grid QDPV systems can achieve complete energy self‐sufficiency, generating, storing, and managing electricity without reliance on external sources, even under diverse environmental conditions, providing a stable and continuous energy supply at minimal expense.

As space exploration and commercial spaceflight become increasingly prevalent, there is significant interest in renewable and efficient energy generation and harvesting methods. Photovoltaics present an attractive alternative to conventional fuel sources and generators due to their superior qualities discussed previously. Multijunction and silicon solar cells are typically employed for space applications due to their ability to withstand significant temperature variations and radiation exposure. However, as mentioned earlier, the epitaxial fabrication process and the need for monolithic deposition of each layer creates challenges.

The use of NIR CQDPV could be particularly beneficial for PV/B systems. CQD solar cells, especially those capable of deep NIR absorption (e.g., PbS), enable the development of high‐power solar cells that capture a broad range of the visible and NIR spectrum, addressing the limitations of the narrow absorption range in current technologies. Figure 17c illustrates the factors influencing PV/B systems in space applications.^[^ ^218^ ^]^

#### 3.1.2 System Design Considerations

PV/B systems typically face two significant challenges: variable input power and fluctuating temperature.

In conventional charging methods called constant current constant voltage (CCCV), the charging process begins with a constant current until a predefined voltage is reached, then switches to a constant voltage mode, where the voltage is maintained while the current gradually decreases until the battery is fully charged. Controlling PV cells to operate at the maximum power point (MPP) and maintain constant power generation (CPG) under varying conditions is crucial for PV/B systems. Deveci et al.^[^ ^219^ ^]^ developed and modelled a PV system, including a DC/DC converter, using MATLAB simulations. By incorporating components such as MPP tracking, battery charging mechanisms, and PID controllers for constant voltage output, the system effectively maintained a steady DC output voltage while operating at the MPP under changing temperature and irradiation conditions. Additionally, Sangwongwanich et al.^[^ ^220^ ^]^ introduced a novel two‐stage control strategy that broadens the CPG algorithm's operating range to the left of the MPP, helping to minimize power overshoots. These advancements can facilitate the integration of NIR CQDPV into PV/B systems.

Lithium‐ion batteries are the most widely used type of battery; however, they are sensitive to temperature, which impacts their effective operation under extreme temperatures and harsh conditions. This issue is also common in other types of batteries. Therefore, maintaining battery efficiency amid fluctuating temperatures is crucial. The development of electric vehicles has been highly beneficial in addressing this issue. Armenta‐Deu et al.^[^ ^221^ ^]^ described adaptive thermal management methods, that adjust dynamically based on real‐time ambient temperature fluctuations to keep the battery within its optimal operating range. Using phase change materials around battery cells helps absorb and release heat, stabilizing temperatures during sudden environmental changes. Additionally, integrating passive and active circuits that switch between cooling and heating systems helps manage extreme temperatures. Lu et al.^[^ ^222^ ^]^ recommended optimized charging protocols to prevent battery overstressing and predictive control systems that anticipate temperature changes, enabling proactive adjustments to thermal management strategies. Additionally, coating techniques, battery pack design, and material engineering including the use of cold/heat‐tolerant electrolytes,^[^ ^223^, ^224^, ^225^, ^226^ ^]^ can also be employed in PV/B systems to enhance their performance in extreme temperatures. These strategies contribute to improved thermal stability and operational efficiency under varying environmental conditions.

**Figure 18:**
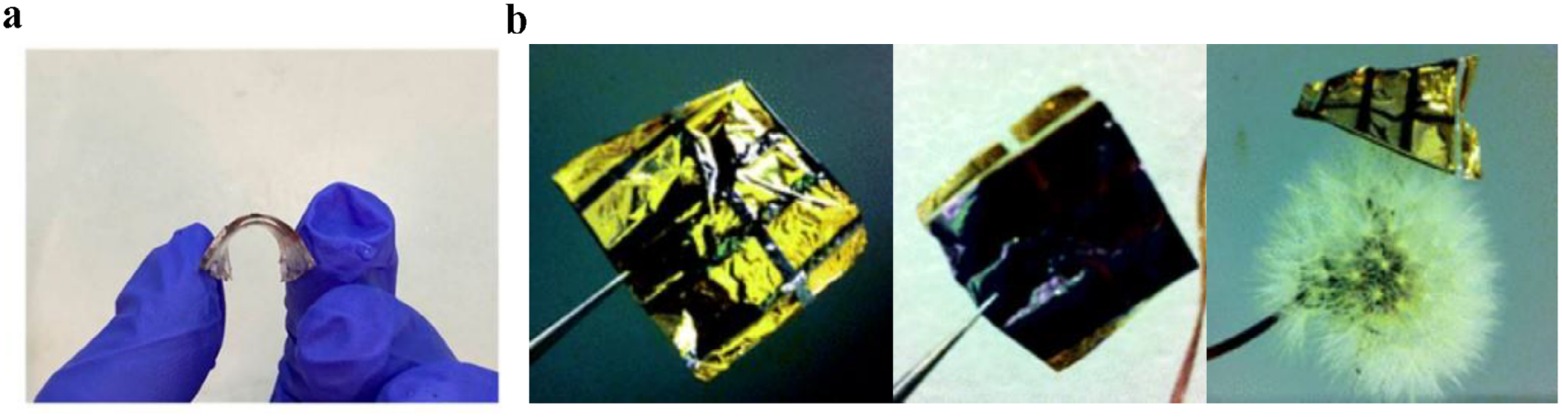
a) The flexibility of an OSC. Reproduced from, Ref. [227]. b) Back side and front side of the flexible solar cell, and the flexible solar cell under the extreme compression state. Reproduced from Ref. [106].

At the same time, PVs based on CQDs allow for flexible, thin‐film applications, making them suitable for varied system designs that can adapt to different surface geometries. For example, Li et al.^[^ ^227^ ^]^ explored the application of flexible and dissolvable OPV based on hydrogel as shown in Figure 18a, which shows equal PCE to their glass substrate‐based counterparts. Zhang et al.^[^ ^106^ ^]^ have successfully demonstrated a flexible PbS CQDPV as shown in Figure 18b.

This can be applied to various surfaces, significantly expanding the potential locations where PV systems can be installed to capture sunlight. This is particularly useful for wearables and portable electronics.

#### 3.1.3 Miniaturized Energy Solutions

As QCD can be fabricated using low temperature processing, without the need of advanced manufacturing techniques, the substrate in which the device is manufactured is not limited to crystalline structures. Instead, textiles and plastics may be used, thus opening the possibility to further miniaturized energy solutions. One such example of overcoming the limited area scaling of silicone photodetectors is presented by Liang et al.^[^ ^228^ ^]^ By fabricating a PbS CQD photodetector on a PEN (poly (ethylene 2,6‐naphthalate)) flexible substrate of 1 cm^2^ area, the miniaturized energy source could be used to measure a heartbeat through the index finger. Even after 200 bending cycles, the output photocurrent decreases only slightly by less than 10%.

Xiao et al.^[^ ^229^ ^]^ also examine the application of PbS CQD solar cells to wearable energy storage devices. PbS PV was fabricated upon a flexible PEN substrate with the top‐performing device reaching a power conversion efficiency (PCE) of 4.92%, with an open‐circuit voltage (*V*_OC_) of 0.56 V, a short‐circuit current density (*J*_SC_) of 22.8 mAcm^−2^, and a fill factor (FF) of 0.41. By integrating the solar cell into a hybrid energy harvesting circuit (HEH) comprising of a rectification circuit and capacitor, the HEH was able to power a digital wristwatch for 5 s.

#### 3.1.4 Compact Integration

A large benefit of nonepitaxial solar cells is their ease of manufacturing and relative integration into integrated systems (health monitoring, lab on chip applications etc). This concept goes beyond the conventional emphasis on “low‐cost” solutions often associated with QCD and solution‐processable electronics. Instead, it emphasizes the unique potential of CQD solar cells to meet demands and deliver performance where crystalline solar cells (e.g Silicon) counterparts face limitations.

### 3.2 Materials for NIR Photovoltaic‐Battery Systems

#### 3.2.1 Photovoltaic Materials

Various CQDs have been studied and demonstrated to have NIR absorption within the NIR range, making them potentially useful for NIR PV/B systems. The lowest bandgaps for commonly used semiconductor CQD materials are presented in Table 8.

**Table 8:** Summary of the materials for NIR PVs.

Composition		Lowest bandgap/eV	Refs.
Lead‐Based CQDs	Lead sulfide (PbS)	0.4	^[^ ^230^ ^]^
	Lead selenide (PbSe)	0.26	^[^ ^231^ ^]^
Non‐Lead CQDs	Copperindium sulfide (CIS)	1.55	^[^ ^232^ ^]^
	Copper indium selenide (CISe)	1.04	^[^ ^233^ ^]^
	Silver sulfide (Ag_2_S)	0.96	^[^ ^234^ ^]^
Other semiconductor CQDs	Mercury telluride (HgTe)	0	^[^ ^235^ ^]^
	Tin sulfide (SnS_2_)	2.18	^[^ ^236^ ^]^
	Tin selenide (SnSe_2_)	1.07	^[^ ^236^ ^]^
Alloyed CQDs	Lead sulfide/ Selenide (PbS_x_Se_1‐x_)	0.26–0.4	^[^ ^237^ ^]^
Quaternary CQDs	Copper indium gallium sulfide (CuInGaS_2_)	2.0	^[^ ^238^ ^]^

#### 3.2.2 Battery Materials

A common argument made by sceptics of renewable energy and more specifically solar cells, often point out that in the case of no incident radiance to the solar cell (on cloudy days or at night) no power is generated. Although valid to some extent for PV modules as stand‐alone entities, the monolithic integration of battery/ energy storage modules has become an imperative part in pushing PV technology forward, to more commercial application as such to reliably and readily provide energy for times at which demand is high.

#### 3.2.3 Lithium‐Based Batteries

##### Li‐ion Batteries

Lithium‐Ion batteries are an attractive method of energy storage due to their inherently high energy density and associated fast response times. As such, integrating Li‐Ion batteries with QDPVs holds promise to more competitive renewable energy sources.

One such example of integrating QDPVs with Li‐Ion batteries was shown by Baek et al.^[^ ^239^ ^]^. By integrating a NIR PbS QDPV with a Li‐ion battery in a wearable wristband, at 1 sun irradiance; a *V*_OC_, *J*_SC_, FF, and PCE of 0.65 V, 24.36 mA.cm^−2^, 63.49% and 10.05% was measured respectively. As a result of the self‐charging capability of the integrated QDPV and Li‐Ion battery, the strap's battery (4.3 V, 40 mAh) reached a full charge in approximately 50 h under 1 sun illumination and around 8 h under concentrated near‐infrared (911.5 nm) light.

Simultaneously, theoretical simulations have been conducted to explore advancements in energy storage and power conversion. Yu et al.^[^ ^240^ ^]^ proposed a hybrid system incorporating a three‐stage charging strategy that achieved an average charge controller efficiency of 96.25%. This method enhances energy capture and prolongs battery life under changing environmental conditions, indicating its potential effectiveness in practical solar energy applications.

##### Alternative Ion‐Based Materials

It is worth further exploring other potential energy storage mechanisms that could be integrated with QDPV and non‐epitaxial semiconductor energy capture devices.

##### Lithium–Sulfur (Li‐S) Batteries

Lithium–Sulphur (Li–S) batteries are an attractive option for PV energy storage devices. Typically, Li–S batteries have higher energy density as compared to conventional Li‐Ion batteries (due to their high sulfur content). Additionally, Li–S batteries are relatively cheaper to manufacture, due to the abundance of sulfur as compared to pure lithium.

The use of Li–S batteries as a storage device with perovskite PVs was demonstrated by Chen et al.^[^ ^241^ ^]^ By using a joint carbon electrode, the Perovskite PV and Li–S battery could be fabricated as one standalone structure. The efficient integration of both PV and Li–S battery resulted in a 5.14% conversion efficiency (from Solar energy absorbed by PV to electrical/chemical/electrical energy conversion by the battery).

##### Sodium‐Ion and Potassium‐Ion Batteries

Sodium/Potassium‐Ion batteries offer better relative stability and life cycle as compared to Li‐Ion batteries. Additionally, the availability of sodium and potassium, as compared to lithium means that manufacturing costs are lower. Hoefler et al.^[^ ^242^ ^]^ proposed the integration of organic photovoltaics (OPVs) with Li‐Ion and Na‐ion batteries. Resulting in an overall efficiency (light to external load via an integrated storage battery subsystem) ranging from 1% to 1.4% for the Li‐Ion system and between 0.4% and 0.6% for the Na‐Ion system. Furthermore, more recently Kin et al.^[^ ^243^ ^]^ demonstrate overall efficiencies of 13.1%–14.4% for OPV/Na‐Ion integrated devices when used for applications in indoor lighting conditions.

##### Flow Batteries

Solar flow batteries have received significant attention as an alternative energy storage device, and as such have benefitted largely from significant leaps in technology development of recent years. Solar flow batteries, in principle, integrate solar energy capture, chemical energy storage, and electrical power delivery into a single system. These devices operate as flow batteries, where energy is stored by dissolving two chemical components in separate liquid electrolytes. These electrolytes are stored in external tanks and are pumped through the system, with a membrane separating the two solutions to enable controlled ion exchange. The integration of solar energy capture into this flow battery structure allows solar flow batteries to harness sunlight directly, bypassing the need for separate photovoltaic (solar) panels.^[^ ^244^ ^]^

Recently, Li et al demonstrated the integration of perovskite/silicon tandem solar cells and flow batteries as an alternative energy capture/storage device.^[^ ^245^ ^]^ The design principles for solar cells to meet requirements of the flow battery must be a sufficient photovoltage, high power conversion efficiency (PCE), and strong corrosion resistance when exposed to aqueous electrolytes. Through optimization methods the integrated solar cell – flow battery device exhibited: an open circuit voltage *V*_OC_ of 1.71 V; short circuit current *J*_SC_ of 15.8 mA/cm^2^; fill factor of 78.3%, and PCE of 21.1%. Such integrated device achieved solar‐to‐output electricity efficiency of 20.1%.

## 4 Efficiency and Energy Conversion Challenges and Future Directions

### 4.1 Power Conversion Efficiency (PCE)

The overall performance of (NIR) CQDPV cells, especially their PCE and long‐term stability, can be influenced by several factors, including but not limited to the stability of CQD materials, surface passivation, and the charge transport layers. Efficiency loss can also occur due to photobleaching and temperature degradation.

The stability of NIR CQDs is the primary factor influencing the efficiency of PV cells. Certain CQDs are susceptible to photodegradation under continuous illumination, which could significantly lower the PCE. Additionally, exposure to oxygen and moisture leads to oxidation, further affecting cell performance. Shi et al.^[^ ^246^ ^]^ explored how ambient water impacts PV performance, showing a PCE reduction from 10.2% to 5.2% when water was present. Photodegradation continues to limit the practical use of CQDs.^[^ ^247^, ^248^ ^]^ Changes in temperature can shift the bandgap of semiconductor materials, influencing the range of photons they can absorb. Materials with smaller bandgaps may capture lower‐energy photons but tend to be more prone to thermal losses when temperature increases.^[^ ^249^ ^]^ Temperature also impacts photoluminescence (PL), and PCE can decrease due to Auger recombination and Shockley Read Hall recombination both increase as temperatures rise.^[^ ^250^, ^251^, ^252^ ^]^ Singh et al.^[^ ^253^ ^]^ reported that nonradiative traps become more significant with increasing temperature, resulting in thermal quenching and a reduction in the radiative recombination efficiency, ultimately reducing the performance of CQDs. Controlling the temperature resistance and stability of QDSCs are critical for their long‐term performance and commercial viability. Core/Shell engineering strategies is one of the most popular practical and research‐backed strategies. For example, Singh et al.^[^ ^253^ ^]^ studied the NIR QDs with a thicker ZnS shell, showing improved photostability under continuous UV light irradiation and with increasing temperature. At the same time, the research on stable shell materials like ZnSe and CdS, gradient alloyed shells like CdSe/ZnSe/ZnS also reduce the lattice mismatch and thermal stress.^[^ ^254^ ^]^ Surface ligand engineering is another method to increase thermal resilience and stability. By replacing the long‐chain organic ligands with shorter and more thermally robust ligands (e.g., 3‐mercaptopropionic acid) or inorganic ligands with less volatile and higher charge transport mobility (e.g., halides, chalcogenide complexes). Meanwhile, cross‐linked ligands can also be used to form a stable ligand network. Ko et al.^[^ ^255^ ^]^ demonstrated that coating QDs with a thiol‐terminated block copolymer can significantly improve resistance to heat (e.g., sustained photoluminescence after thermal exposure at 100 °C) and chemical oxidation under ambient or oxidant‐rich conditions. Encapsulation is also widely used for stability improvement and temperature resistance. Inorganic encapsulation (e.g., SiO_2_, TiO_2_, and Al_2_O_3_) can block the oxygen and moisture. Kim et al.^[^ ^256^ ^]^ demonstrated a solar cell with a light‐harvesting layer of silica‐embedded CdSe–ZnS QDs, which exhibited no loss of quantum yield, increased device efficiency by ∼23%, and maintained performance for over 168 h in iodide/triiodide electrolytes. At the same time, glass–glass encapsulation with edge sealing and flexible encapsulants (e.g., PMMA, PVP) for flexible devices are also critical for long‐term outdoor operation.

However, increasing PCE and maintaining long‐term operational stability are often competing priorities, which requires strategic trade‐offs.

Efficiency‐focused direction usually prioritizes enhance the PCE by increasing the light absorption, charge carrier extraction, and spectral utilization. Techniques such as MEG, tandem/multijunction architectures, optical light management (e.g., plasmonic nanostructures, antireflective coatings) can significantly raise PCE, but can also increase fabrication complexity and may introduce new failure modes such as parasitic absorption in nanostructures or interfacial degradation in multijunction stacks. On the opposite side, stability‐focused solutions primarily target at materials interfaces and environmental resilience such as UV exposure, moisture, temperature variation. As mentioned before, ligand engineering, passivation methods can improve the performance and protect against the degradation, however, may also limit light absorption or introduce interfacial barriers that lower charge extraction efficiency.^[^ ^160^, ^182^ ^]^

There is an inherent conflict in optimizing CQD layers: improving their ability to absorb light and generate charge can make them more susceptible to environmental damage, while enhancing their durability might hinder charge transport or increase energy losses. To overcome this, a multiobjective optimization approach is needed. Hybrid methods—such as combining halide passivation with light‐managing films, which aims to preserve performance improvements while maintaining stability. Meanwhile, the development of machine learning and artificial intelligence^[^ ^257^ ^]^ offers new opportunities to achieve a balance between efficiency and robustness.

In conclusion, achieving commercial success for CQD NIR PVs and photodetection systems requires an integrated design strategy that addresses both stability and performance.

### 4.2 Energy Storage Efficiency

The main problems affecting the energy storage efficiency includes the energy losses during the charging and discharging, charge/discharge rate limitations, conversion losses, self‐discharge, and cycle life and degradation.

#### 4.2.1 Battery Bank

The power output from a solar PV energy system and the AC loads of household appliances are managed by controlling the charging and discharging of the battery bank. The state of charge (SOC) of the battery at any given moment is represented by the following expression,^[^ ^258^ ^]^ which means that the state of charge at time *t* is equal to the initial state of charge, plus the cumulative energy added to the battery (considering charging efficiency), minus the cumulative energy discharged (considering discharging efficiency):

$$SOC\left(t\right)=SOC\left(0\right)+\eta_{c}\sum_{k=0}^{t}P_{CB}\left(k\right)+\eta_{d}\sum_{k=0}^{t}P_{DB}\left(k\right)$$

Here, *SOC*(0) represents the initial state of charge of the battery at *t* = 0, *P_CB_* is the electrical power being charged into the battery, *P_DB_* is the electrical power discharged from the battery bank, and η_*c*_​ and η_*d*_​ denote the charging and discharging efficiencies, respectively.

#### 4.2.2 Converter

The converter shown in Figure 17 is connected between DC/AC buses. Its function is to convert the DC current produced by the PV system and battery bank (during discharge) into AC for the load. The efficiency (η_Inv_) of the converter is assumed to be constant and depends on the relationship between power input and output:

$$P_{\mathrm{InvOut}}=P_{\mathrm{InvIn}}\eta_{\mathrm{Inv}}$$

For the off‐grid solar PV system:

$$P_{\mathrm{InvIn}}=P_{\mathrm{PV}}+P_{\mathrm{DB}}$$

For the grid‐tied solar PV system:

$$P_{\mathrm{InvIn}}=P_{\mathrm{PV}}$$

Both experimental studies and theoretical simulations are conducted to enhance energy storage efficiency. For instance, investigations explore improvements in battery materials, optimization of charging and discharging cycles, and the development of more efficient power management strategies. Kell et al.^[^ ^259^ ^]^ utilized a deep reinforcement learning algorithm to optimize battery charging and discharging in homes equipped with PV systems, discovering that system performance theoretically improved with larger battery sizes, thereby reducing grid dependency. F. Wang^[^ ^260^ ^]^ introduced an analytical model designed to predict and optimize battery discharge performance by analyzing various discharge behaviors in battery electrodes. From experimental aspects, Hirt et al.^[^ ^261^ ^]^ enhanced Lithium‐ion battery charging systems by improving model predictive control through Bayesian optimization of its parameters. Additionally, Kollimalla et al.^[^ ^262^ ^]^ introduced a novel control strategy aimed at effectively managing battery charge and discharge rates within a hybrid energy storage system that includes both batteries and supercapacitors. This approach helps to reduce battery stress, optimize energy usage, and extend the lifespan of the battery.

### 4.3 Environmental and Safety Concerns

Most materials used in PV manufacturing especially for NIR PV, are potentially toxic and may be released into the environment via air and water and cause some serious problems. Similarly, battery production often involves heavy metals that could lead to environmental pollution if not properly recycled. Therefore, they should be monitored throughout the system's lifetime, which aims to minimize the environmental impacts by reducing waste during manufacturing and recycling at the end of their lifetime. For example, Bayer et al.^[^ ^263^ ^]^ managed to decrease the concentration of toxic cadmium by up to 10 times by adjusting the temperature and solution concentration during the deposition process of CdS thin films. However, the most widely used CQD materials for NIR PV cells are lead‐chalcogenide compositions, as indicated in Table 6. The synthesis and ligand‐exchange processes for these materials require significant amounts of organic solvents, which can pose health risks if inhaled excessively or for individuals who handle these materials over extended periods. Liu et al.^[^ ^264^ ^]^ developed a direct synthesis method for PbS ink by optimizing the synthesis process, which reduces the potential exposure to harmful chemicals.

Although CQD NIR PVs systems offer exciting potential due to their flexibility and tunability, they also have environmental and lifecycle challenges that must be thoroughly evaluated to ensure sustainable commercialized. As mentioned before, the heavy meatal enable board NIR absorption and high efficiencies, but also raise toxicity and environmental contamination concerns, particularly during manufacturing, device aging, and end‐of‐life disposal. Therefore, maintaining high stability and performance directly impact the lifecycle environmental footprint.

Compared with conventional epitaxial semiconductors, solution growth and fabrication of CQDs, which requires lower temperature and simpler equipment can achieve lower energy consumption. This also contributes positively to the lifecycle environmental footprint.

From a systematic perspective, CQD PV/B system supports the renewable energy storage and off‐grid applications, which offers a way to reduce dependence on fossil fuels. However, ensuring environmental sustainability requires that future research focus on lead‐free alternatives (e.g., InP‐based CQDs), the development of standardized recycling processes, and the adoption of more eco‐friendly synthesis techniques.

### 4.4 Future Directions

Integrated energy capture and storage devices offer a cheap and efficient solution to problems connected to the intermittent nature of solar energy. Achieving solid state integration between a photovoltaic device and energy storage system would address this.^[^ ^265^ ^]^ In recent years the ‘Photo‐rechargeable battery’ has developed through significant performance improvements. One such example is by Wang et al.^[^ ^266^ ^]^ using MA_2_Bi_2_I_9_ as the photoelectric conversion and energy storage active material layer. The cells exhibited PCE of 1.13% (*J*_SC_ = 6.62 mA/cm^2^; *V*_OC_ = 0.573 V; FF = 29.79%) as a photovoltaic, and was able to illuminate external LEDs when 10 photo modules were connected in series. The overall conversion efficiency (discharge energy divided by incident light energy) was 0.05%.

When assessing whether zinc‐ion batteries were suitable for solid state integration with PV devices, Boruah et al.^[^ ^267^ ^]^ achieved photo charge conversion efficiencies of around 1.8% when using MoS2/ZnO photocathodes as the active material. This work showed the novel capability to both harvest and store energy within the same material, without the need for complicated integration of both a PV device and battery together in solid state.

In addition to zinc storage devices, lithium based solid state devices have showed promise for applications to dual energy capture/ storage. As demonstrated by Xu et al.,^[^ ^268^ ^]^ by integrating four individual CH_3_NH_3_PbI_3_ perovskite solar cells (PSCs) in series, they were able to directly photocharge lithium‐ion batteries (LIBs) comprising a LiFePO_4_ cathode and a Li_4_Ti_5_O_12_ anode. The integrated PSC–LIB units achieved a high overall photoelectric conversion and storage efficiency of 7.80%.

Zinc air batteries, as investigated by Fu et al.,^[^ ^269^ ^]^ have shown promising. By developing nitrogen‐substituted graphdiyne (N‐GDY), which features an extensive π‐conjugated carbon network that functions as a photoresponsive dual‐purpose electrocatalyst, sunlight‐driven processes can efficiently transfer photoelectrons into the conduction band of N‐GDY. Such device had a power conversion efficiency of 1.02%.

**Figure 19:**
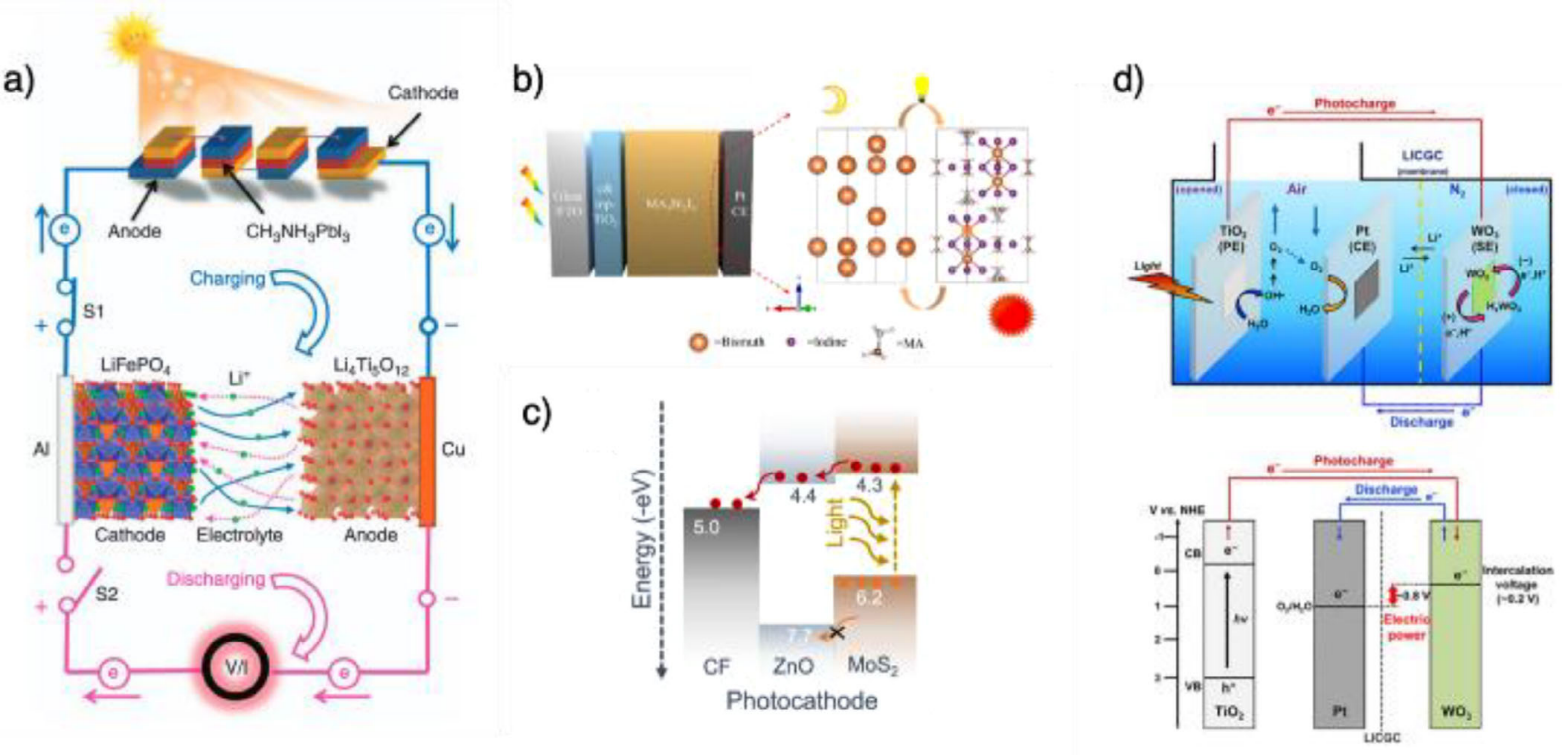
Examples of solid state integrated solar capture and energy storage devices selected from literature a) Lithium based charging, storage, and discharge system developed by Xu et al.^[^ ^268^ ^]^ b) Schematic illustration of the MA_2_Bi_2_I_9_ based system investigated by Wang et al.^[^ ^266^ ^]^ c) Photophysical mechanism of the dual ZnO/MoS_2_ integrated harvesting and storage method demonstrated by Boruah et al.^[^ ^267^ ^]^ d) Schematic illustration of the solar water battery developed by Kim et al.^[^ ^270^ ^]^

Solar water batteries have developed attention in recent years due to photoelectrochemical water oxidation and energy storage use. Kim et al.^[^ ^270^ ^]^ developed a ‘solar water battery’ which operates when light striking the photoelectrode causes the water to be photooxidized, thus charging the battery in one integrated system. Such a device achieved high columbic efficiency of over 90% and demonstrated good charge retention with limited self‐discharge over extended time (showing suitability for storage applications. Examples of listed references are shown in Figure 19.

Artificial intelligence (AI) and machine learning (ML) are accelerating the design and optimization of CQD materials by improving the speed and precision of synthesis and characterization. For example, Voznyy et al.^[^ ^68^ ^]^ optimization has been used to systematically explore reaction conditions, leading to the synthesis of high‐quality PbS QDs. By identifying oleylamine as a growth inhibitor, this method produced nanoparticles with record‐low excitonic linewidths of 55 meV at 950 nm and 24 meV at 1500 nm. ML models are also speeding up the prediction of material properties. Multi‐layer perceptron (MLP) neural networks can accurately predict the electronic and optical properties of QDs in nanocomposites while significantly reducing computational costs.^[^ ^271^ ^]^ In characterization, ML also automates the time‐consuming manual evaluation of QDs as single‐photon sources. Convolutional neural networks (CNNs) have been applied to InAs/GaAs QDs, learning from spectral data to reliably identify the most suitable emitters for quantum applications.^[^ ^272^ ^]^ Furthermore, ML enables inverse design, where a model predicts the physical structure needed to achieve target optical properties. Tandem deep neural networks have been used to predict the layer thicknesses of InP/ZnSe/ZnS QDs with over 99.8% pearson correlation coefficient. These models overcame the common nonuniqueness problem by using both emission and absorption spectra as inputs. The framework was then adapted via transfer learning to design CdSe‐based QDs.^[^ ^273^ ^]^ Together, these data‐driven methods provide powerful tools for navigating the complex parameter spaces of QD engineering, offering greater control over material properties and speeding the path from research to real‐world applications.

Although a promising technology, as outlined above, there remains challenges related to long term stability and optimization of energy conversion efficiencies.^[^ ^274^ ^]^ Additionally, the challenging fabrication process must be developed from a lab‐based methodology to more industrial high‐volume scale. Currently, the most CQD photovoltaic devices are fabricated by spin coating and layer by layer ligand exchange. This is effective in laboratory setting but unsuitable for large‐scale manufacturing due to limitations in throughput, uniformity, and reproducibility.^[^ ^275^ ^]^ For commercial purposes, the fabrication techniques must develop toward scalable, roll‐to‐roll compatible processes such as inkjet printing, spray coating, dip coating, slot‐die coating, and blade coating.^[^ ^276^ ^]^ These QD deposition techniques offer higher material utilization, less resource waste, and better compatibility with flexible substrates, which are critical for the wearable and portable PV/B hybrid systems. Moreover, industrial high‐volume scale also requires overcoming the challenges in CQD film thickness control, interfacial engineering, and multilayer device architecture alignment, all while maintaining device performance and long‐term operational stability.^[^ ^275^ ^]^ The most promising direction for automatic and standardize production of CQDs is combined with the continuous flow synthesis, coupled with in‐line ligand exchange and self‐assembled monolayers.^[^ ^277^ ^]^ At the same time, ensuring the scale‐up process does not affect the uniformity of CQD packing, the carrier mobility across the thin films, or the energy alignment at heterojunctions,^[^ ^275^ ^]^ can bridge the gap between lab‐scale innovation and practical deployment.

## 5 Conclusion

This review has provided an in‐depth discussion on colloidal quantum dots, covering their composition, properties, synthesis methods, and applications in near‐infrared photovoltaics. Additionally, it has introduced photovoltaic‐battery hybrid energy systems for NIR applications, highlighting potential NIR CQD materials, commonly used battery materials, and current technologies for system integration. Future research should include system‐level studies focusing on the multidisciplinary integration of NIR CQDPV/B hybrid energy systems, which may become a key area of exploration. The demand for more reliable, intelligent, and flexible PV/B hybrid energy systems is expected to grow, potentially replacing conventional power generators and collectors. Although laboratory tests have shown excellent performance for both batteries and solar cells, the advancement of PV/B hybrid energy systems is still influenced by the availability and accessibility to high‐performance components. In light of the cross‐disciplinary progress in science and technology, fostering technical exchanges focused on NIR CQDPV/B hybrid energy systems will be essential in the future.

## Conflict of Interests

The authors declare no conflict of interest.

## Data Availability

Research data are not shared.
